# Calnexin Is Essential for Survival under Nitrogen Starvation and Stationary Phase in *Schizosaccharomyces pombe*

**DOI:** 10.1371/journal.pone.0121059

**Published:** 2015-03-24

**Authors:** Andrés Núñez, Dominic Dulude, Mehdi Jbel, Luis A. Rokeach

**Affiliations:** Department of Biochemistry and Molecular Medicine, Université de Montréal, Montréal, Québec, Canada; University of Pittsburgh, UNITED STATES

## Abstract

Cell fate is determined by the balance of conserved molecular mechanisms regulating death (apoptosis) and survival (autophagy). Autophagy is a process by which cells recycle their organelles and macromolecules through degradation within the vacuole in yeast and plants, and lysosome in metazoa. In the yeast *Schizosaccharomyces pombe*, autophagy is strongly induced under nitrogen starvation and in aging cells. Previously, we demonstrated that calnexin (Cnx1p), a highly conserved transmembrane chaperone of the endoplasmic reticulum (ER), regulates apoptosis under ER stress or inositol starvation. Moreover, we showed that in stationary phase, Cnx1p is cleaved into two moieties, L_Cnx1p and S_Cnx1p. Here, we show that the processing of Cnx1p is regulated by autophagy, induced by nitrogen starvation or cell aging. The cleavage of Cnx1p involves two vacuolar proteases: Isp6, which is essential for autophagy, and its paralogue Psp3. Blocking autophagy through the knockout of autophagy-related genes (atg) results in inhibition of both, the cleavage and the trafficking of Cnx1p from the ER to the vacuole. We demonstrate that Cnx1p is required for cell survival under nitrogen-starvation and in chronological aging cultures. The death of the *mini_cnx1* mutant (overlapping S_cnx1p) cells is accompanied by accumulation of high levels of reactive-oxygen species (ROS), a slowdown in endocytosis and severe cell-wall defects. Moreover, mutant cells expressing only S_Cnx1p showed cell wall defects. Co-expressing mutant overlapping the L_Cnx1p and S_Cnx1p cleavage products reverses the death, ROS phenotype and cell wall defect to wild-type levels. As it is involved in both apoptosis and autophagy, Cnx1p could be a nexus for the crosstalk between these pro-death and pro-survival mechanisms. Ours, and observations in mammalian systems, suggest that the multiple roles of calnexin depend on its sub-cellular localization and on its cleavage. The use of *S*. *pombe* should assist in further shedding light on the multiple roles of calnexin.

## Introduction

Cell fate is mainly determined by the balance between conserved molecular mechanisms controlling apoptosis (death) and autophagy (survival) [1]. In developed organisms, apoptosis has a protective role by eliminating superfluous cells, cells infected by pathogens and cells that have suffered non-reparable damages [1,2]. For a long time, apoptosis was the prerogative of higher organisms, but ever-mounting evidences have demonstrated that unicellular organisms like yeasts can undergo apoptotic programs [3,4]. In yeast, apoptotic cells exhibit distinct morphological and biochemical changes such as DNA condensation and fragmentation, induction of metacaspase activity and accumulation of reactive oxygen species (ROS) [5,6,7]. Apoptotic cell death occurs also in chronologically aging cells, and it is associated with the accumulation of ROS [8,9,10].

Autophagy is a conserved cellular process from yeast to mammals, by which organisms recycle their intracellular components into double-membrane vesicles (autophagosomes), which deliver them for degradation within the vacuoles in yeasts and in plants, or to the lysosome in metazoan [11,12,13]. Although autophagy may occur at basal conditions, its importance is revealed under stress situations in which it acts as a survival mechanism [14,15]. Autophagy is highly induced by challenging environmental conditions including nutrient limitation, heat shock and oxidative stress, as well as aging [14,15]. At the molecular level, autophagy is a multistep process involving a highly conserved set of proteins encoded by the so-called autophagy-related genes (*atg*) [11,13]. In the fission yeast *Schizosaccharomyces pombe* several of these proteins (including Atg1, Atg6, Atg8 and Atg9) have been characterized for their role in the regulation of autophagy induced by nitrogen starvation [16,17]. Besides Atg proteins, induction and execution of autophagy requires also a starvation-specific protease designated Isp6 in *S*. *pombe*, which is important for protein degradation and integrity of vacuoles under nitrogen depletion [18]. The expression of *isp6* ^*+*^ was shown to be induced by nitrogen starvation, and Isp6p was first designated as a serine protease based on sequence similarity to the budding yeast vacuolar protease Prb1 [19].

The endoplasmic reticulum (ER) is an organelle specialized in the synthesis of membrane and secretory proteins, and regulates protein folding and trafficking, cellular responses to stress, and intracellular calcium (Ca^2+^) levels [20]. Disturbance of the ER lumen environment by conditions such as alterations in Ca^2+^ homeostasis, inhibition of N-linked glycosylation, and changes in redox status are known to cause ER stress and induce the accumulation of misfolded/unfolded proteins [21]. These ER-stress conditions trigger a compensatory phenomenon called the unfolded protein response (UPR) [21]. The UPR activates the expression of specific genes encoding ER chaperones to support correct protein folding, and causes the inhibition of general protein synthesis to reduce the burden of substrates to be fold in the ER. Failure of the UPR to alleviate ER stress leads eventually to apoptotic cell death. ER stress underlies numerous human pathologies including several neurodegenerative disorders such as Alzheimer’s and Parkinson’s disease, as well as diabetes, ischemia and cardiovascular disease [22].

Calnexin is an ER transmembrane chaperone that participates in protein translocation, protein folding, and the quality control of newly synthesized polypeptides [23]. Calnexin has been implicated in numerous genetic trafficking diseases including cystic fibrosis, and several coagulation disorders [23]. In these diseases, calnexin provokes the ER-retention and the targeting to degradation of proteins that are constitutively misfolded due to inherited mutations [23]. Structurally, calnexin is a type I membrane-bound protein. At the center of the lumenal portion of calnexin there is a highly conserved domain (*hcd*), which is present in all eukaryotic species with the exception of *S*. *cerevisiae* [24]. Calnexin has a large ER lumenal domain, a transmembrane domain (TM), and an ER-retention motif in its cytoplasmic tail.

Mammalian calnexin was reported to participate in apoptosis triggered by tunicamycin treatment, a potent inhibitor of protein *N*-glycosylation and inducer of ER stress [25,26,27]. In fact, initial indications as to the role of calnexin in apoptosis came from experiments in *S*. *pombe*. Torgler et al. [28], first showed that *S*. *pombe* calnexin (Cnx1p) is required for cell death caused by heterologous expression of mammalian pro-apoptotic protein BAK. We showed that overexpression of Cnx1p causes apoptotic cell death [29]. Moreover, the use of mutants demonstrated that Cnx1p regulates apoptosis induced by ER stress or by inositol starvation [29,30].

In this study, we examine the involvement of calnexin in nutrient-stress responses. We show that Cnx1p is processed into two moieties, L_Cnx1p and S_Cnx1p. This proteolytic cleavage is dependent on vacuolar proteases Isp6 and Psp3 and autophagy. The viability of cells expressing truncated versions of Cnx1p is severely reduced under nitrogen starvation and in chronologically aging cells. The truncation mutant mini_Cnx1p cells, corresponding to the cleavage product S_Cnx1p, exhibits severely reduced viability, high levels of the ROS apoptotic marker and cell-wall defects. Interestingly, co-expression of mutants overlapping both L_Cnx1p and S_Cnx1p cleavage products restores viability to wild-type levels, as well as reverses the ROS-accumulation and the cell-wall defect phenotype. Taken together, our results demonstrate that Cnx1p is required for survival under nitrogen starvation and in stationary phase, indicating that this ER protein plays a significant role during the cellular responses to these nutritional changes.

## Materials and Methods

### Yeast strains and media

*S*. *pombe* strains used in this study are listed in S1 Table. Mutants strains were obtained by mating, transformation (lithium acetate method; [31]), purchased from *Bioneer Corporation* (Daejeon, Korea), *YGRC* (Yeast Genetic Resource Center, Japan), or kindly provided by other groups. Unless otherwise stated, cells were grown at 30°C in Edinburgh Minimal Medium (EMM) with 2% of glucose, 5g/L glutamate as nitrogen source and required supplements for each strain [32].

### Expression vectors

#### Construction of Isp6p and Psp3p expressing plasmids

Plasmids pREP41*isp6-HA* and pSLF272*psp3-HA* were constructed in order to obtain strains expressing Isp6p and/or Psp3p under the control of mid-strength, thiamine-repressible promoter of pREP41. Paired oligonucleotides Psp3-ATG:(5'-TAATAACTCGAGTCATGAGAGTTTCTTGGATTAGCGGTCTC-3'), and Psp3-Stop: (5'-TTATTAGCGGCCGCCCTCATAATTGTTGAAGGCAAGGACATTAGGTGTGTC CTCAGG-3'); Isp6-ATG: (5'- TAATAACTCGAGACATGAGAATTCCTTATTCAA ATCTTTTTTCTGCC-3'), and Isp6-Stop: (5'-TTATTAGCGGCCGCCTTCTTGAGCA CC ATTGAAAGCGAGAAGGTTAATAGTGCTGCTACC-3'), were used to amplify by PCR the open reading frame (ORF) of each gene, using *S*. *pombe* genomic DNA as template. Purified PCR products were digested with *Xho*I and *Not*I and cloned into pSLF272 [33]. This vector contains the *ura4* ^+^ gene as selectable marker and allows to fuse the HA epitope to the 3'-end to the cloned sequence. To obtain pREP41*isp6-HA*, the fragment between *Sph*I and *Sal*I in pSLF272*isp6-HA* was cloned into plasmid pREP41 (with *LEU2* gene of *S*. *cerevisiae* as marker; [34]).

#### Construction of plasmids expressing Cnx1-Venus fusions

The pREP41*cnx1-Venus* plasmid was constructed by a three-piece ligation that simultaneously introduced in frame both the Cnx1p and the Venus coding sequences into the pREP41 digested with *Sal*I-*Bam*HI restriction sites. The coding sequence of *cnx1* ^*+*^ was obtained by PCR amplification from the genomic DNA of WT *S*. *pombe* SP247 using the primers containing the restrictions sites (underlined) *Sal*I and *Spe*I: Cnx1-ATG-Sal1: (5’-GCCGTCGACATGAAGTACGGAAAGGTATCTTTTCTAGC-3’);and Cnx-Spe1: (5’-GTTTACTAGTGTCTTCATTCTTCGCAGTTGGTG-3’). The coding sequence of yellow fluorescent protein Venus variant was obtained by PCR amplification from the plasmid pCS2-Venus [35]. The primers used contained the restriction sites (underlined) *Spe*I and *BamH*I: Venus-Spe1 (5’-GCCACTAGTGTGAGCAAGGG CGAGG-3’); and Venus-STOP-BamHI: (5’-GCCGGATCCTTACTTGTACAGCTC GTCCATGCCG-3’). All truncated versions of the full-length pREP41*cnx1-Venus* construct were obtained by subcloning into this plasmid digested *Sal*I-*Spe*I. The coding sequences of *lumenalTM_cnx1*, *lumenal_cnx1* were obtained by PCR amplification from the genomic DNA of WT *S*. *pombe* SP247 using the following primers: for *lumenalTM_cnx1* Cnx1-ATG-Sal1and Lum-TM-spe1 (5’-GTTTCT CGAG AG CAA AG AAATAAAAGTAACA-3’), and for *lumenal_cnx1* Cnx1-ATG-Sal1 and Lum-Spe1 (5’-GTTTACTAGTCCCAATTTCAGGAGTCTCG-3’). The coding sequences of *Δhcd_cnx1* and *mini_cnx1* were amplified using the primers Cnx1-ATG-Sal1 and Cnx-Spe1 with the plasmids pREP41*mini_cnx1* and pREP41*Δhcd_cnx1*as template, respectively [36]. All Venus fusions were proven to sustain viability in the *cnx1Δ* background.

#### Other plasmids

Details for plasmid constructions for the expression of truncated versions of Cnx1p were previously published [29,36]. Plasmid expressing GFP-Atg8 (modified pTN54 derived from pREP41) was a kind gift from Dr. Korou Takegawa (Kyushu University, Japan).

### Protein extraction and immunoblotting

Cell extracts were prepared by lysis with glass beads in an immunoprecipitation buffer (50 mM HEPES pH 7, 50 mM NaCl, 1 mM CaCl_2_, 1% Nonidet P40), containing 10 mM iodoacetamide, 1 mM PMSF and leupeptin 1 ng/ml, plus specific protease and phosphatase inhibitor cocktails for fungal and yeast extracts (*Sigma-Aldrich*, P8215). After quantification by the Bradford method, protein extracts were fractionated on 8%- 12% (w/v) SDS-PAGE gels. Proteins were transferred onto nitrocellulose membranes according to the manufacturer's instructions. Suitable antibodies were used to detect Cnx1p (anti-Cnx1p rabbit polyclonal antibody LAR223), Cnx1-myc (with anti-myc, mouse mAb 9E10), Isp6-HA (with anti-HA, mouse mAb 12CA5), GFP-Atg8 (with anti-GFP, *Invitrogen*) and Tubulin (anti-tubulin, *Sigma-Aldrich*).

### Time-course for the processing of calnexin and GFP-Atg8

Cell samples were taken at different time points from growing cultures. Time 0 corresponds to an OD_600_ equivalent to 0.1 resulting from a 1:10 dilution from cells in exponential phase. Samples were taken every 24 h and protein extracts were obtained and analyzed by immunoblotting as described above.

### Isp6 protease activity assay

Isp6 activity in cell extracts was determined using Azocoll (*Calbiochem*) as substrate. The procedure was adapted from the Proteinase B (PrB) Azocoll assay [37,38]. Briefly, 100 mg of Azocoll was prewashed twice with 50 ml of PBS under agitation for 2 h, centrifuged each time 10 min at 10,000X g and resuspended at 2.5 mg/ml in PBS. Cells collected each day (about 20 OD_600_) for the time-course for the processing of calnexin experiment described above, were lysed with glass bead in 100 mM Tris-HCl pH 7.6 buffer without protease inhibitors. Fifty μg of protein extract diluted in 200 μl of 100 mM Tris-HCl pH 7.6 were incubated with 800 μl of prewashed Azocoll with end-to-end agitation for 2 h at 28°C. The undigested Azocoll reagent was precipitated by centrifugation at 15,000X g for 10 min and the absorbance of the supernatant was read at 520 nm. The protease activity is presented as the OD_520_ of the incubation supernatant. Each sample was tested between 3 to 6 times and two experiments were performed.

### Autophagy-induction assays

For autophagy-induction assays, strains were grown in supplemented EMM (glutamate as nitrogen source) to exponential phase (OD_600_≤ 0.8). Cells were washed twice with water and resuspended in EMM-N (EMM without glutamate or any other nitrogen source). Cell samples were taken at the indicated points, usually 0, 4 and 8 hours after the shift into EMM-N. Immunoblotting with suitable antibodies (as described above) was performed to analyze the processing of GFP-Atg8 and of Cnx1p.

### Fluorescence microscopy analyses

To analyze the cellular localization of the wild-type Cnx1-Venus fusion and the Cnx1p truncated derivatives fused to Venus: lumenalTM-Cnx1-Venus, lumenal_Cnx1-Venus, Δhcd_Cnx1-Venus, and mini_Cnx1-Venus, liquid cultures were seeded to an OD_600_~0.1 in EMM media and then grown to mid-exponential phase (OD_600_~0.5). Following which, exponential cells were either maintained to the stationary phase (day 3) or washed with water and switched to nitrogen starvation medium (EMM-N) for 24 hours. Cells were incubated with the fluorescent dye FM4–64 to label vacuoles and with the fluorescent dye Hoechst 33342 to label the nucleus (both from *Molecular Probes*). Typically 1 ml of growing cultured cells was pulsed with 16μM FM4–64 for 30 minutes. The cells were then washed, resuspended in fresh EMM and then chased for 30 minutes to allow all the dye to reach the vacuole. During the chase phase, the staining of the nucleus was performed by adding Hoechst 33342 at 16μM. Following which, the cells were analyzed by fluorescence microscopy using a fluorescence inverted microscope (Nikon TE2000U). The cell samples were analyzed by using a ×1,000 magnification with the following filters: 450–490 nm (FITC) for the Venus fusions proteins, 540–580 nm (Texas-Red) for the FM4–64 vacuolar staining and 335–383 nm (DAPI) for DNA staining with Hoechst 33342. Cell fields shown in this study are representative of experiments repeated at least three times. Images were acquired using a motion-picture camera, CCD coolSnapFX M 12 bit (Roper Scientific, Tucson, AZ), and treated with UIC Metamorph software.

For Calcofluor staining of cell-wall material (CSM) analysis, about 4x10^6^ cells starved for nitrogen were harvested, resuspended in 3μl of Calcofluor white (50μg/ml; *Sigma-Aldrich*, F-3543), applied on a microscope slide and covered with a coverslip. Cells were observed as above with a UV filter. CSM positive cells were counted from image processed with Image J software (NIH). Experiments were performed three time and at least 300 cells were counted each time.

### Endocytosis assays

Cells grown to mid-exponential phase in EMM or from nitrogen starvation (EMM-N) cultures for 48 hours were kept on ice for 5 minutes, subsequently combined with the FM4–64 dye at a final concentration of 16μM and immediately analyzed by fluorescence microscopy at indicated time points until the dye reaches the vacuoles (as described in [39]).

### Viability assays

#### Survival under nitrogen starvation

For viability assays under nitrogen starvation, strains were grown in supplemented EMM (glutamate as nitrogen source) to early exponential phase (OD_600_≤ 0.6). After two washes with water, cultures were diluted to OD_600_ = 0.3 in 30 ml of fresh medium EMM-N (without glutamate nor any other nitrogen source) and split into two cultures (duplicates). To measure clonogenicity, cell samples were plated onto supplemented EMM plates every 24h for 6 days, and viability was determined as cfu/ml after 5 days incubation at 30°C. Hundred % viability was set as the maximum cfu/ml reached for each strain before decline (usually 24h after the medium shift). Experiments were repeated at least three times.

#### Survival in chronologically aging cultures

Chronological lifespan assay (CLS) was assessed by CFU (colony-forming units) counting as previously described [8,9]. Briefly, *S*. *pombe* strains were streaked on EMM plates and grown for 3 days at 30°C. Cultures were inoculated in EMMC (EMM supplemented with all non-selective amino acids at 112mg/L) until it reached OD_595_ 3–4, diluted and grown until OD_595_ 5–6, then diluted again and grown to the maximal density. At this point, culture samples were diluted and plated every 24h onto EMM plates for the first five days and every 2–3 days thereafter. CFU were counted after incubating the plate at 30°C for 5 days. The beginning of the CLS curve (100% viability) corresponds to the day with the highest CFU, indicating the end of the exponential phase and the beginning of stationary phase. Each experiment was repeated at least three times with duplicate samples.

#### Determination of intracellular reactive oxygen species (ROS)

For the analysis of intracellular ROS, the superoxide sensitive probe Dihydroethidium (DHE, *Molecular Probes*) was used, as described previously [9]. Briefly, 4x10^6^ cells were harvested, incubated in PBS with 10μM DHE for 30 min at 30°C. Cells were centrifuged, resuspended in PBS, and 10,000 cells were analyzed by flow cytometry using a BD FACS Calibur (488-nm laser and emission on FL-2 channel 560–600 nm). The percentage of positive stained cells was determined as the population of fluorescent cells with higher fluorescence intensity than an unstained negative control. To account for the proportion of dead cells that can accumulates over time in the sample and eventually reduce the absolute number of ROS-positive cells, for each measurement an aliquot of cells were also stained with Phloxin B in parallel as previously described [9], and ROS-positive cells were thus plotted per viable cells. Each experiment was repeated at least three times.

## Results

### The processing of calnexin is dependent on the vacuolar proteases Isp6 and Psp3

**Fig 1:**
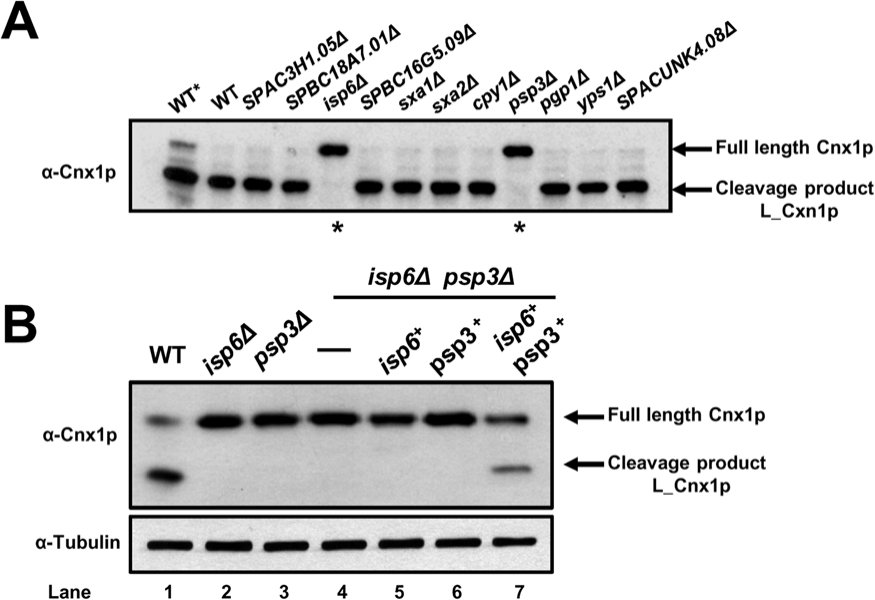
The processing of calnexin is dependent on the Isp6 and Psp3 proteases. **(A)** *Screening to identify proteases involved in the cleavage of calnexin*. Single protease-mutants (see Table 1) and WT cells (SP247) were grown in EMM until 3 days in stationary phase. Cells lysates were prepared in absence of protease inhibitors; incubated 30 min at 30°C for full protease action, and Cnx1p was detected by immunoblotting with anti-Cnx1p antibodies (α-Cnx1p). Lane 1 (WT*) corresponds to an exponential phase sample. To prevent inhibition of proteases cascades cells were lysed in absence of protease inhibitors. We hypothesized that in this manner the only inhibition of cleavage to be observed would be due to the genetic knockout of the specific protease tested. Cell lysates were subjected to SDS-PAGE and the cleavage of Cnx1p was analyzed by western blotting. **(B)** *The expression of both the Isp6 and Psp3 proteases is necessary for the processing of calnexin*. WT strain (SP247), single mutants *isp6Δ* (SP18041) and *psp3Δ* (SP18174), and double knockout *isp6Δpsp3Δ* (SP18417) expressing pREP41*isp6-HA* (SP18419), pSLF272*psp3-HA* (SP18421) or both plasmids (SP18423), were grown in EMM w/o thiamin to stationary phase. Cell lysates were prepared in presence of protease inhibitors and Cnx1p was detected by immunoblotting with anti-Cnx1p antibodies (α-Cnx1p). The detection of tubulin with anti-tubulin antibodies was used as loading control (α-Tubulin). The positions of full-length Cnx1p and the L_Cnx1p cleavage product are indicated on the left.

Previously, we have shown that Cnx1p is proteolytically cleaved under normal growth conditions, and this processing is enhanced when cells reach stationary phase [30]. This cleavage results in two fragments of calnexin: i) a large one comprising most of the lumenal part of the protein (L_Cnx1p); and ii) a smaller one (S_Cnx1p) encompassing the last 40–50 residues of the lumenal portion, the TM domain, and the cytosolic tail [30]. The factors involved in the physiological cleavage of calnexin remain unknown. To begin elucidating the physiological role of processing of Cnx1p, we decided to identify the protease(s) responsible for its cleavage. Eleven proteases (listed in Table 1) were selected as candidates according to functional and cellular location criteria [40,41]. Knockout mutants and WT cells were grown in minimal medium (EMM), samples were taken after 3 days in stationary phase. To prevent inhibition of protease cascades, cells were lysed in absence of protease inhibitors. We hypothesized that in this manner that the only inhibition of cleavage observed would be due to the genetic knockout of the specific protease tested. Cell lysates were subjected to SDS-PAGE and the cleavage of Cnx1p was analyzed by western blotting. A lysate of a WT strain grown in exponential phase was used as reference for full-length Cnx1p (Fig. 1A, WT*). As shown in Fig. 1A, no cleavage was observed in the *isp6Δ* (SPAC4A8.04) and the *psp3Δ* (SPAC1006.01) null-mutants, indicating that these proteases are involved in the processing of calnexin. Interestingly, both Isp6p and Psp3p are localized within the vacuole [11,42] indicating they could collaborate in the cleavage of Cnx1p, and suggesting that the processing takes place in this compartment. It is noteworthy, that the knockout of other vacuolar proteases tested had no effect on the processing of Cnx1p.

**Table 1:** Proteases tested in this study.

Strain	Systematic ID	Standard Name	Status	Product	Localization
SP18174	SPAC1006.01	*psp3* ^+^	Published [43]	Serine protease	Vacuole
SP18041	SPAC4A8.04	*isp6* ^+^	Published [43]	Serine protease	Vacuole
SP17176	SPCC1795.09	*yps1* ^+^	Published [43]	Aspartic protease	Cell surface
SP17198	SPAC26A3.01	*sxa1* ^+^	Published [44]	Aspartic protease	Cell surface
SP17199	SPAC1296.03c	*sxa2* ^+^	Published [43,44]	Serine Carboxypeptidase	Cytosol/extracellular
SP17220	SPAC3H1.05	*—-*	Biological role inferred	CAAX prenyl protease	ER membrane
SP17221	SPBC18A7.01	*—-*	Biological role inferred	X-pro dipeptidase (predicted)	ER membrane
SP17175	SPCC1259.10	*pgp1* ^+^	Published [43]	Metallopeptidase	Mitochondrion
SP17260	SPAC19G12.10c	*cpy1* ^+^	Published [45]	Carboxypeptidase	Vacuole
SP17222	SPBC16G5.09	*—-*	Unknown	Serine carboxypeptidase (predicted)	Vacuole
SP17223	SPACUNK4.08	*—-*	Biological role inferred	Dipeptidyl peptidase (predicted)	Vacuole

To clarify the role of each protease in Cnx1p cleavage, we overexpressed Psp3p and Isp6p, singly and in concert, in a double *isp6Δpsp3Δ* background. As shown in Fig. 1B, lane 4, the cleavage of Cnx1p is completely blocked in the absence of both proteases. Moreover, no full-cleavage product of Cnx1p is observed when either Isp6 or Psp3 alone is expressed (Fig. 1B, lane 5, 6). The presence of the full-cleavage product of Cnx1p is only observed when Isp6p and Psp3p are co-expressed in the cell (Fig. 1B, lane 7). These data demonstrate that both proteases, Isp6p and Psp3p, are required for the physiological processing of calnexin in *S*. *pombe*.

### The processing of calnexin is concomitant with the activation of autophagy

**Fig 2:**
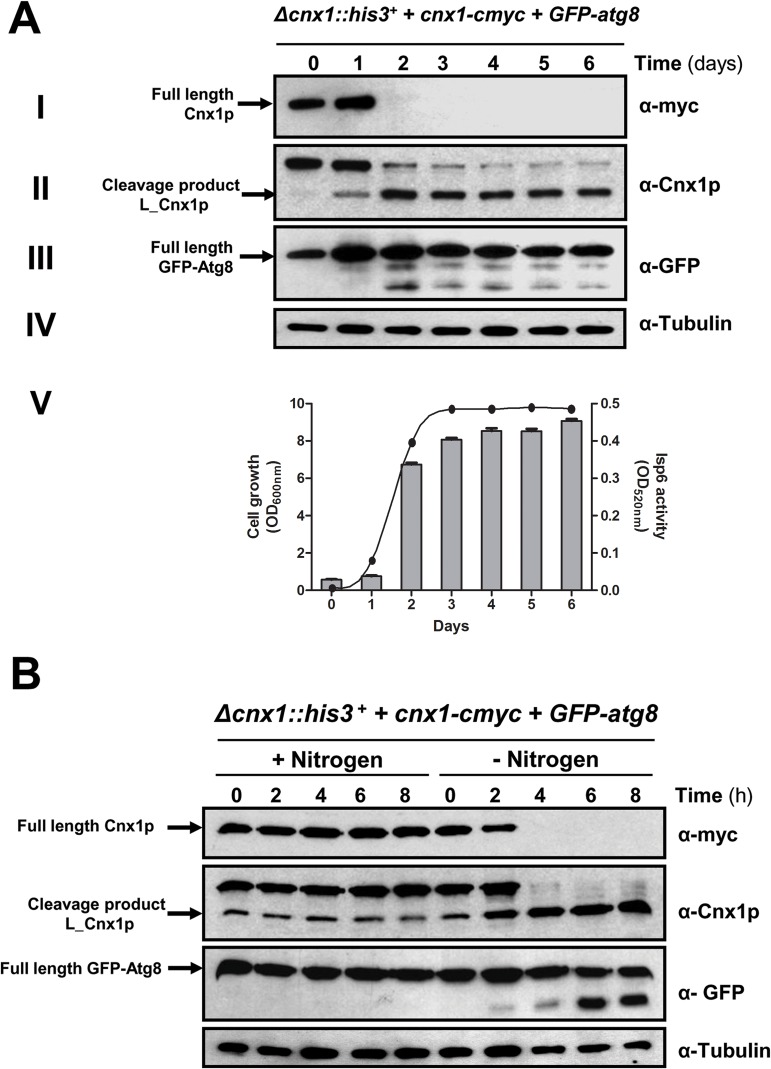
The processing of calnexin is concomitant with the induction of autophagy. **(A)** *Processing of calnexin*, *GFP-Atg8 and Isp6-HA during normal growth*. Strain *Δcnx1*::*his3* ^*+*^+ pREP42*cnx1-cmyc* + pTN54*GFP-atg8* (SP18147) was cultured in minimal medium (EMM) and samples were taken every 24 h from early exponential phase to late stationary phase. Cell extracts were processed and submitted to immunoblotting analysis as follows: (I) Cnx1p-cmyc was detected with anti-cmyc monoclonal antibodies (α-myc). (II) Cnx1p-cmyc was detected with anti-Cnx1p antibodies (α-Cnx1p). (III) GFP-Atg8 was detected with anti-GFP antibodies (α-GFP). (IV) Tubulin (loading control) was detected with anti-tubulin antibodies (α-Tubulin). (V) The activity of Isp6 (histogram, right Y-axis) during the cell growth phase (curve, left Y-axis) of the strain SP18147 was determined using a proteinase assay with Azocoll substrate. The positions of full-length Cnx1p and the L_Cnx1p cleavage product are indicated on the left. **(B)** *Nitrogen starvation triggers the processing of calnexin*. Exponential culture of the strain *Δcnx1*::*his3* ^*+*^+ pREP42*cnx1-cmyc* + pTN54*GFP-atg8* (SP18147) in EMM was shifted to minimal medium without nitrogen (EMM-N). Cell samples were taken every 2 hours, and cell extracts were analyzed by immunoblotting. The processing of calnexin was monitored with anti-cmyc (α-myc) and anti-Cnx1p (α-Cnx1p) antibodies. The induction of autophagy was detected by following the cleavage of the autophagy marker GFP-Atg8 with anti-GFP antibodies (α-GFP) in immunoblots. Tubulin (as loading control) was detected with anti-tubulin antibodies (α-Tubulin). The positions of full-length Cnx1p and the L_Cnx1p cleavage product are indicated on the left, as well as the position of the full-length GFP-Atg8 fusion.

The cleavage of Cnx1p is enhanced in stationary phase [30], when the lack of nutrients leads to reorganization in cellular metabolism to sustain viability for extended periods of time. Previous studies have reported that Isp6p is an essential vacuolar protease for autophagy and for survival in stationary phase [18]. Furthermore, mRNA levels of *isp6* ^*+*^ are under the influence of culture medium [11], suggesting that its expression is sensitive to the availability of nutrients. Since autophagy is induced in stationary phase when nutrients are limited [46,47], and because Isp6p participates in the cleavage of Cnx1p, we decided to study the possibility that the processing of Cnx1p is linked to autophagy. Atg8p is a protein essential for autophagy, and its processing occurs with the induction of this process [17]. As such, the cleavage GFP-Atg8 is used as a marker for induction of autophagy in yeast and mammalian systems. Accordingly, the cleavage of both, Cnx1p and GFP-Atg8 was assessed at different time points using the strain SP18147, which expresses Cnx1p-myc and GFP-Atg8 from two different plasmids. Cells were cultured in EMM from early exponential phase (OD_600_ = 0.1) to 3 days after stationary phase was reached. The cleavage of GFP-Atg8 occurs around day 2, when cells are close to stationary phase and the processing of Cnx1p is increased (Fig. 2A, panels I-III). We have also used the c-myc C-terminal tagged version of calnexin to follow its processing as the loss of the detection of the c-myc epitope. This indicates that the cleavage of Cnx1p and the activation of autophagy are aligned in time during growth.

Isp6p, like its homolog in *S*. *cerevisiae* Prb1p, is processed post-translationally to become active [48,49,50]. In order to monitor Isp6p activation during the cell growth phase, we measured its activity in cell extracts using the Azocoll-hydrolysis assay [37,38]. The Isp6p activity was minimal during first days and sharply increased at day 2 (Fig. 2A, panel V). Azocoll degradation was specific to Isp6p in this assay since no activity was detectable in *isp6*-null cells (data not shown). The activation of Isp6p is concomitant with the processing of Cnx1p and the cleavage of GFP-Atg8 observed from day 2. Taken together these observations and the results in Fig. 1A suggest that Isp6p is involved in the cleavage of calnexin in *S*. *pombe* during the cell’s adaptive response to stationary phase.

As nitrogen starvation is a powerful inductor autophagy [16,17], we next studied the processing of Cnx1p under these conditions. Strain SP18147 was grown in EMM to exponential phase (OD_600_≤0.5). Half of the culture was shifted to EMM-N (EMM without any source of nitrogen) to induce autophagy, keeping the other half in EMM as a control. Samples were taken at different time points after the shift to EMM-N and analyzed by immunoblotting to study the cleavage of Cnx1p. As shown in Fig. 2B, the processing of Cnx1p is triggered after 4 hours in nitrogen depletion, coinciding with the cleavage of GFP-Atg8. These results further confirm that the processing of calnexin correlates with the activation of autophagy in *S*. *pombe*. It is noteworthy that the processing of GFP-Atg8 is weaker in stationary phase as compared to nitrogen depletion, thus suggesting that the level of induction of autophagy in stationary phase is weaker than in nitrogen starvation, however sufficient for the processing of calnexin.

### Autophagy regulates the processing of calnexin

**Fig 3:**
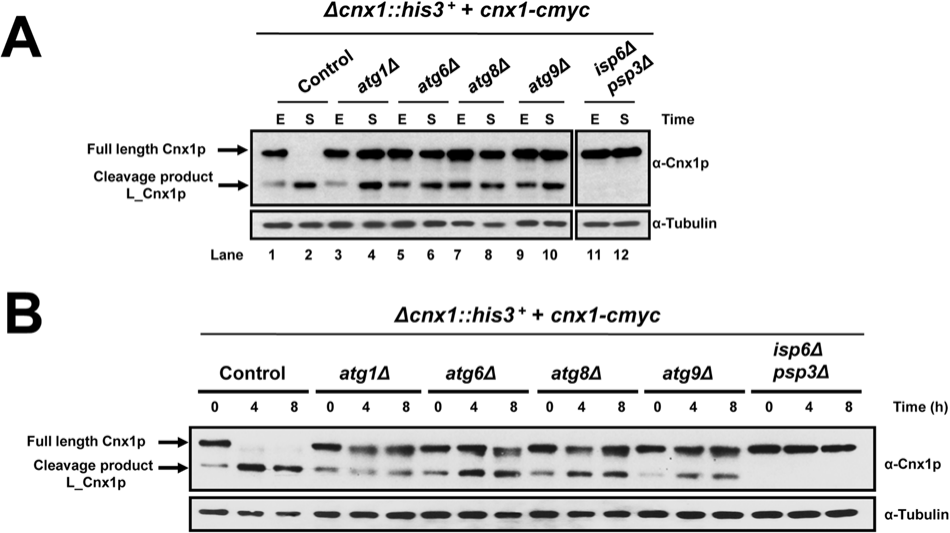
The processing of calnexin is defective in autophagy-null mutants. **(A)** *Analysis of the cleavage of Cnx1p in stationary phase*. Strains *atg1Δ* (SP18084), *atg6Δ* (SP18087), *atg8Δ* (SP18086), *atg9Δ* (SP18414), *isp6Δpsp3Δ* (SP18340) and control cells (WT, SP18077) were grown in EMM and samples were taken at exponential phase (E), and stationary phase for 3 days (S). Cnx1p and tubulin (loading control) were detected by immunoblotting with anti-Cnx1p or anti-tubulin, respectively. **(B)** *Analysis of the cleavage of Cnx1p in nitrogen starvation*. Strains *atg1Δ* (SP18084), *atg6Δ* (SP18087), *atg8Δ* (SP18086), *atg9Δ* (SP18414), *isp6Δpsp3Δ* (SP18340) and control cells (WT, SP18077) were grown in EMM until early exponential phase (OD_600_<0.5), washed twice with water and resuspended in EMM-N. Samples at times 0, 4 and 8 hours after the shift were taken. Cnx1p was detected by immunoblotting with anti-Cnx1p (α-Cnx1p). Tubulin (loading control) was detected with anti-tubulin (α-Tubulin) antibodies. The positions of full-length Cnx1p and the L_Cnx1p cleavage product are indicated on the left.

The core of the autophagy process involves proteins highly conserved from yeast to mammals [12,13]. To investigate a possible mechanistic involvement of autophagy, we analyzed whether the cleavage of Cnx1p in stationary phase is altered in different autophagy-defective backgrounds: *atg1Δ*, *atg6Δ*, *atg8Δ* and *atg9Δ*. These genes are essential for autophagy by acting at different steps during this process [11,13]. Samples after 3 days in stationary phase were taken to study the processing of Cnx1p by immunoblotting. The cleavage of Cnx1p in the *atg*-mutant backgrounds was clearly reduced during stationary phase as compared to control cells, as depicted in Fig. 3A. Whereas in the control there is no observable full-length Cnx1p band, in the corresponding *atgΔ* lanes there is considerable amount of residual full-lenth Cnx1p (lane 2 vs lane 4, 6, 8, 10). The observation that *atg-*mutants did not block completely the cleavage of Cnx1p suggests that other cellular pathway(s) are also involved in the regulation of this activity. Tor1, Sck2 and Sty1 are signaling proteins involved in nutrient sensing and the adaptive response to nitrogen starvation [9,51,52]. We therefore analyzed whether the processing of Cnx1p was affected in absence of these proteins by using knockout strains. No significant defect in processing of Cnx1p was observed (data not shown).

Next, we analyzed whether the processing of Cnx1p occurs under nitrogen starvation in *atg* mutants. Here again, autophagy-null mutants showed severe inhibition in the processing of Cnx1p (Fig. 3B), however the cleavage was not entirely blocked. The same result was obtained by analyzing double *atg*-mutants (data not shown).

Taken together, these results show that the processing of calnexin in *S*. *pombe* is regulated by autophagy, however other cellular pathway(s), yet to be determined, are also involved.

### The sorting of calnexin to the vacuole is impaired in autophagy-defective mutants

**Fig 4:**
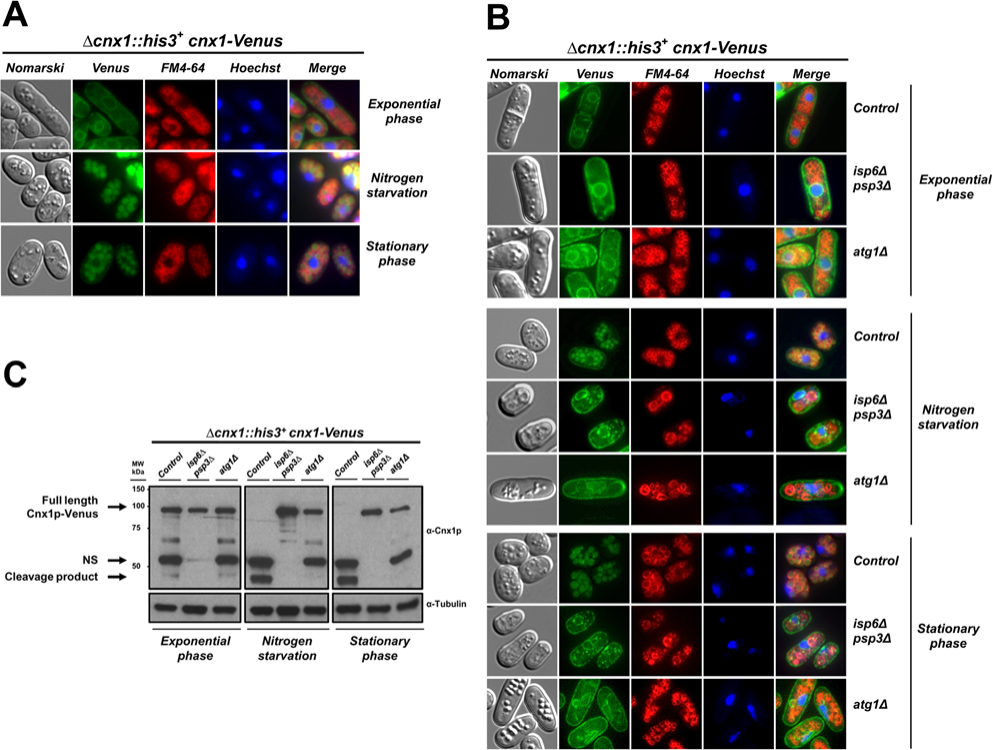
The targeting of calnexin to the vacuole is impaired in both, the *atg1Δ* and the *isp6psp3Δ* backgrounds. (**A)** *Cellular localization of the Cnx1-Venus fusion*. Strain *Δcnx1*::*his3* ^*+*^ *+* pREP41*cnx1-Venus*(control, SP19201) was grown in EMM to mid-logarithmic phase. The culture was split into two, half was maintained until stationary phase for 3 days, and the other half was shifted to EMM-N medium to induce nitrogen starvation (24 h). Cells were analyzed by fluorescence microscopy for the localization of Cnx1-Venus, FM4–64 was used as a marker for the vacuole, and Hoechst33342 was used as a marker for the nucleus. Nomarski bright-field microscopy was used to monitor cell morphology. Merged images were used to determine colocalization. **(B)** *The localization of Cnx1-Venus is defective in the atg1Δ and the isp6Δpsp3Δ backgrounds*. Cultures of strains *Δcnx1*::*his3+ Δatg1*::*KanMX4 +* pREP41*cnx1-Venus* (SP19197), *Δpsp3*::*KanMX4 Δisp6*::*ura4* ^*+*^ *Δcnx1*::*his3* ^*+*^ *+* pREP41*cnx1-Venus* (SP19209) and *cnx1Δ*::*his3* ^*+*^ *+* pREP41*cnx1-Venus* (control, SP19201) were grown in EMM and treated as described in (A). **(C)** *Analysis of the cleavage of Cnx1-Venus*. Cell samples from the experiment described in (B) were taken and analyzed by immunoblotting. Cnx1-Venus and tubulin (loading control) were detected with anti-Cnx1p (α-Cnx1p) or anti-tubulin (α-Tubulin), respectively. The positions of full-length Cnx1-Venus and its cleavage products are indicated on the left. NS, non-specific band.

The vacuole is the central organelle where autophagy takes place, providing proteases and the appropriate environment for the degradation of the imported cargo into more simple compounds for the subsequent syntheses required for cell survival [53]. Our results show a link between the processing of Cnx1p, autophagy and the vacuolar proteases Isp6p and Psp3p, suggesting that the processing of Cnx1p takes place in the vacuole. To test this hypothesis, we fused the Venus fluorescent protein to the C-terminus of Cnx1p (Cnx1-Venus; see Materials and Methods), to study its cellular localization under different conditions. The Cnx1-Venus fusion is functional as it complements the knockout of the *cnx1* ^*+*^ gene, which is essential for viability. Fig. 4A shows that Cnx1-Venus is localized within the ER during exponential phase, as previously determined by Elagöz et al. [36]. Interestingly, the Cnx1-Venus fusion colocalizes with the vacuolar marker FM4–64 in cells in stationary phase or after 3 days under nitrogen starvation conditions. The subcellular redistribution of the Cnx1-Venus also correlates with the complete processing of the fusion protein observed by immunoblotting (Fig. 4C, control lane in the Nitrogen starvation and Stationary phase versus Exponential phase panel).

Next, we wondered whether the defective processing of Cnx1p observed in *atg*-mutants was due to its impaired sorting to the vacuole. To address this point, we analyzed the localization of Cnx1-Venus in the *atg1Δ* background in which autophagy is blocked [11,13] and Cnx1p processing is reduced. We also studied the localization of Cnx1-Venus in the *isp6Δpsp3Δ* background, in which the cleavage is completely blocked. In exponential phase, the ER-distribution of Cnx1-Venus was the same in both, control cells and the mutants (Fig. 4B). Cnx1-Venus did not co-localize within the vacuole (FM4–64 marker) in the *atg1Δ* mutant when cells have reached the stationary phase or under nitrogen depletion, but the fusion remained in the ER (Fig. 4B). However, as shown in Fig. 4C (and see Fig. 3A), calnexin is processed to a certain extend in *atg*-mutants, this may indicate that a certain pool of Cnx1p reaches the vacuole but is undetectable by fluorescence microscopy, and/or it is cleaved in an intermediate compartment close to the ER.

These observations indicate that the targeting of Cnx1p in *atg*-mutants is defective, as is its processing. Intriguingly, the trafficking of Cnx1-Venus to the vacuole in *isp6Δpsp3Δ* mutant was also impaired under the same conditions (Fig. 4B), suggesting that these proteases may be involved in general sorting from the ER to the vacuole. As expected, the processing of the Cnx1p fusion is impaired in the *isp6Δpsp3Δ* background (Fig. 4C).

To assess if the targeting of Cnx1p to the vacuole is specific to this protein or a part of a general trafficking of the ER to the vacuole, we studied the trafficking of the ER-resident protein Sec61 (Sec61p-GFP) or the artificially ER-retained GFP (Venus-ADEL). Similarly to Cnx1p-Venus when cells reaches stationary phase or are submitted to nitrogen depletion, Sec61p-GFP or and ER-retained GFP localize to the vacuole (see S1 Fig.). This suggests that the trafficking of Cnx1p-Venus and other ER proteins to the vacuole is a common feature under these conditions [54].

### Different regions of calnexin are required for survival under nitrogen starvation

**Fig 5:**
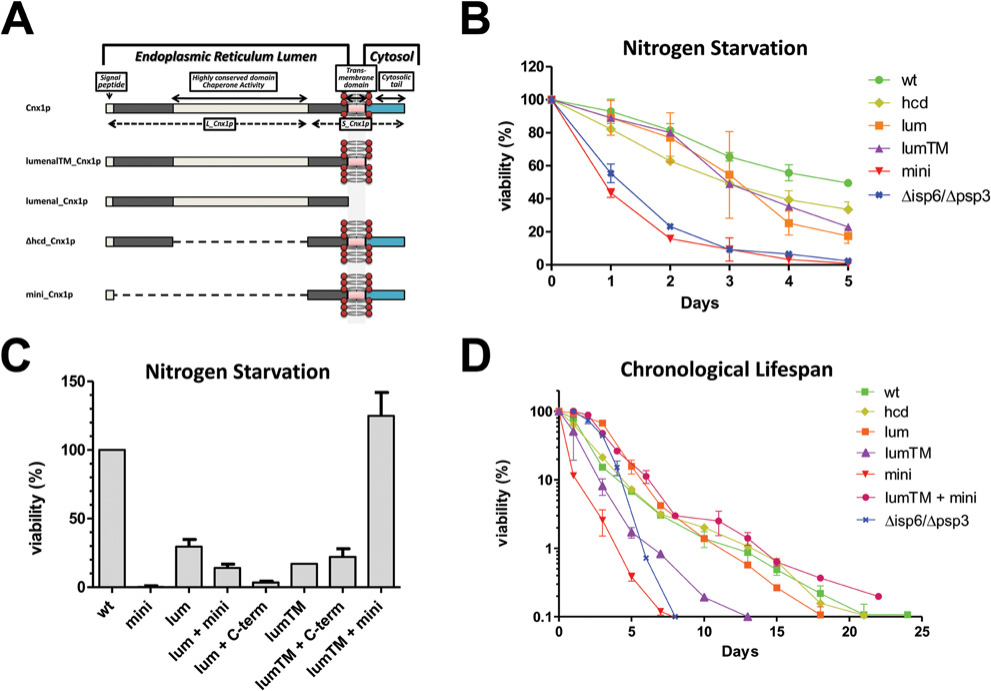
Different regions of calnexin are required for survival under nitrogen starvation and in stationary phase. **(A)** *Schematic representation of wild-type calnexin (Cnx1p) and truncated mutants*. The Large (L_Cnx1p) and small domain (S_Cnx1p) obtained after the processing of calnexin are depicted in the full length version. **(B)** *Viability under nitrogen starvation*. Strains *mini_cnx1* (SP18344), *lumenal_cnx1* (18346), *lumenalTM_cnx1* (SP18348), *Δhcd_cnx1* (SP18350), *isp6Δpsp3Δ* (SP18340) and WT control cells (SP18342) were grown in EMM until early exponential phase (OD_600_<0.5), washed twice with water and resuspended in EMM-N. Culture samples were plated every 24h onto EMM, and incubated for 5 days at 30°C. CFU/ml was determined and 100% viability was set as the maximum CFU/ml reached for each strain before decline (usually 24h after the shift). **(C)** *Co-expression of lumenalTM_Cnx1p rescues the rapid loss of viability of mini_cnx1 under nitrogen depletion*. The viability of the strains, presented as relative to WT defined as 100%, *mini_cnx1* (SP18344), *lumenal_cnx1* (SP18346), *lumenal_cnx1* + *mini_cnx1* (SP18282), *lumenal_cnx1* + *C-term_cnx1-Venus* (SP18300), *lumenalTM_cnx1* (SP18348), *lumenalTM_cnx1* + *C-term_cnx1-Venus* (SP18303), *lumenalTM_cnx1* + *mini_cnx1*(SP18285) and control cells (SP18342) was analyzed as described in (B). **(D)** *Analysis of chronological lifespan (CLS) in calnexin mutants*. Strains *mini_cnx1* (SP18344), *lumenal_cnx1* (SP18346), *lumenalTM_cnx1* (SP18348), *Δhcd_cnx1* (SP18350), *lumenalTM_cnx1* + *mini_cnx1* (SP18285), *isp6Δpsp3Δ* (SP18340) and WT control cells (SP18342) were cultured in EMM supplemented with all the amino acids, except those required for selection (EMMC). Culture samples from different days (within a period of 25 days) were plated onto EMM and incubated at 30°C for 5 days. CFU/ml was determined and 100% was set as the maximum CFU/ml reached for each strain. Y-axis is shown in logarithmic scale.

That the cleavage of Cnx1p occurs under nitrogen deprivation (Fig. 2B) suggests that its processing may be part of the adaptive response to this nutritional stress. To test this possibility, we analyzed the viability for cells expressing truncated versions of Cnx1p under nitrogen starvation. The Cnx1p truncation mutants used are schematically represented on Fig. 5A. Viability was analyzed by plating and counting colony-forming units (cfu) at different time points after the shift to minimal medium without nitrogen (EMM-N). The mutant *mini_cnx1* (encompassing the 52 residues in the lumenal domain + TM + cytosolic tail) loses viability in a remarkable manner under nitrogen deprivation (Fig. 5B). The vast majority of cells did not form colonies after 5 days under nitrogen starvation. Interestingly, the death kinetics of *mini_cnx1* is similar to that of the autophagy-defective strain *isp6Δpsp3Δ*. Moreover, this death is most likely specific to nitrogen-starvation since WT and Cnx1p mutants have similar growth rate and viability in exponential phase (see S2A, S2C Fig. and S2 Table). These results with *mini_cnx1* suggest that the lumenal portion of Cnx1p (containing the chaperone and lectin activities) is required for the cell’s response under nitrogen depletion. However, the mutant *Δhcd_cnx1* lacking the chaperone and lectin activities has better viability than *mini_cnx1* indicating that other functions encoded by the lumenal portion of Cnx1p are required for survival under nitrogen starvation. The mutant *lumenal_cnx1* (encompassing the whole lumenal domain, but lacking the TM and cytosolic domain) and the mutant *lumenalTM_cnx1* (lumenal domain + TM) also exhibit reduced viability compared to WT (Fig. 5B), albeit to a lesser extent than *mini_cnx1*. These results indicate that the C-terminal part of Cnx1p is also important for survival under nitrogen starvation.

Nitrogen deprivation induces the processing of Cnx1p yielding two fragments, L_Cnx1p and S_Cnx1p. The experiments described above imply that both portions of Cnx1p are important for cell survival in the absence of nitrogen. To examine this point, we hypothesized that it could be possible to recover viability at Cnx1p WT levels by co-expressing two different Cnx1p truncation mutants overlapping the two moieties resulting from Cnx1p processing (Fig. 5C). Remarkably, cell viability in *mini_cnx1* is completely rescued by co-expressing *lumenalTM_cnx1*, but no recovery of viability is observed by co-expressing *lumenal_cnx1* (Fig. 5C). Together, these data suggest that the lumenal and the cytosolic tail of calnexin are required to maintain viability under nitrogen deprivation, and that both parts must be attached to the membrane through the TM for an efficient response to this nutritional stress.

### Different regions of calnexin are required for survival in stationary phase

Chronological cell aging, similar to nitrogen starvation, involves the recycling of cellular constituents for survival through autophagy [14]. This common point led us to investigate whether Cnx1p could play a role in chronological lifespan (CLS) in *S*. *pombe*. The CLS of strains expressing different truncated versions of Cnx1p varied significantly (Fig. 5D). As expected, the autophagy-deficient mutant *Δisp6Δpsp3* exhibited short CLS compared with control cells (Fig. 5D). Concurring with the results obtained under nitrogen starvation, *mini_cnx1* shows a remarkably short CLS, and this phenotype is entirely rescued by co-expression of the *lumenalTM_cnx1* mutant (Fig. 5D). The Cnx1p mutants and WT showed similar growth rate and viability in exponential phase in rich EMMC medium (see S2B, S2D Fig. and S2 Table) indicating that the loss of viability observed is specifically related to chronological aging and not a general defect reducing strains fitness accumulated during logarithmic growth.

**Fig 6:**
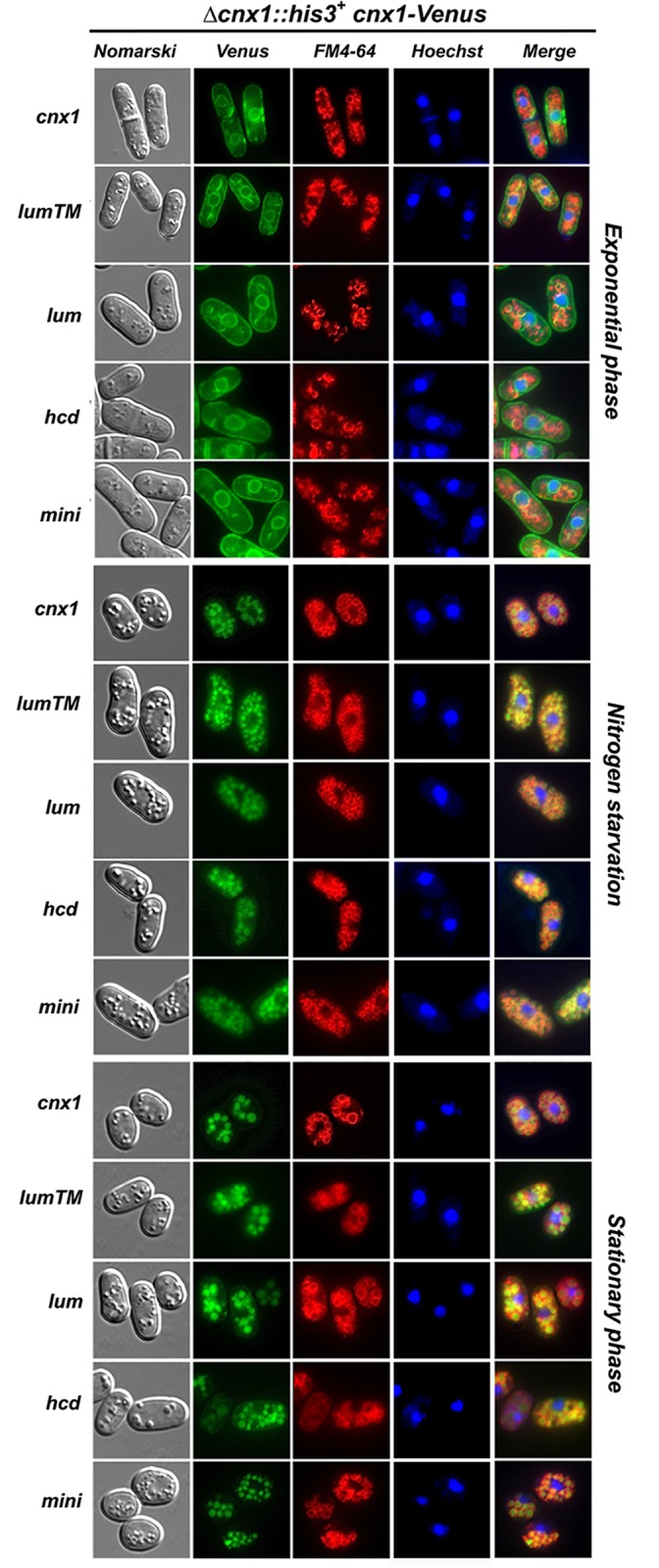
The processing and the ER-to-vacuole trafficking of calnexin and its mutant versions occur under nitrogen starvation and in stationary phase. *Subcellular localization of calnexin-truncated mutants fused to Venus*. Strains *cnx1*::*his* ^*+*^ *+* pREP41*lumenalTM_cnx1-Venus* (SP19207), pREP41*lumenal_cnx1-Venus* (SP19174), pREP41*Δhcd_cnx1-Venus* (SP19211), pREP41*mini_cnx1-Venus* (SP19212) and pREP41*cnx1p-Venus* (control, SP19201) were grown in EMM to mid-logarithmic phase. The culture was split into two, half was maintained until stationary phase for 3 days, and the other half was shifted to EMM-N medium to induce nitrogen starvation (24h). The cells were analyzed by fluorescence microscopy for Venus for the localization of Cnx1-Venus), FM4–64 was used as a marker for the vacuole, and Hoechst 33342 was used as a marker for the nucleus. Nomarski bright-field microscopy was used to monitor cell morphology. Merged images were used to determine colocalization.

*S*. *pombe isp6Δpsp3Δ* and *atg1Δ* mutants show low viability under nitrogen starvation and after reaching stationary phase (Fig. 5B, 5D; [16]). Furthermore, these mutants are defective in the processing and sorting of Cnx1p to the vacuole (Fig. 4). Thus, we wondered if the loss of viability observed in Cnx1p mutants could be due to their impaired trafficking. To address this point, we fused the Venus fluorescent-protein marker to the C-termini of the truncated versions of Cnx1p (see Materials and Methods), and we studied their localization by microscopy. The mutants analyzed (lumenal_Cnx1-Venus, lumenalTM_Cnx1-Venus, Δhcd_Cnx1-Venus and mini_Cnx1-Venus) displayed the same cell localizations as the full-length fusion, Cnx1-Venus, under both, nitrogen depletion and stationary phase (Fig. 6). Cell samples from these experiments were taken and analyzed by immunoblotting to verify whether processing does occur. The mutants were processed as the wild-type fusion under the conditions examined (S3A and S3B Fig.). These results show that the loss of viability observed in cells expressing some truncated version of Cnx1p is not caused by a defective localization or processing of the fusion proteins.

Together, these observations indicate that calnexin in *S*. *pombe* plays key roles in survival under nitrogen starvation as well as in determining CLS. Moreover, different parts of calnexin appear to play different or complementary functions in survival under both conditions.

### High levels of ROS accompany the death of *mini_cnx1* cells under nitrogen starvation and chronological aging

**Fig 7:**
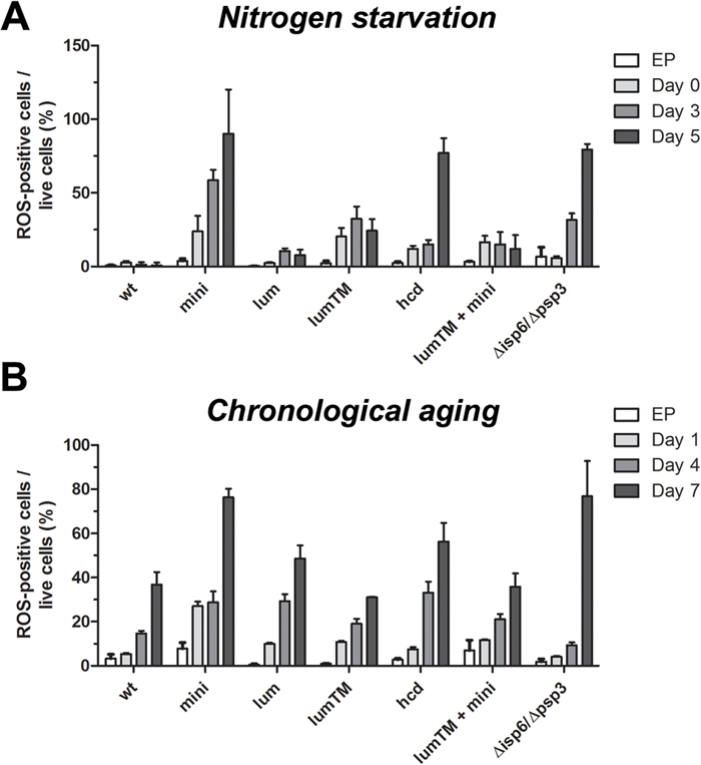
High levels of ROS accompany the death of *mini_cnx1* cells under nitrogen starvation and chronological aging. Accumulation of ROS in cells under nitrogen starvation **(A)** and **(B)** during aging cultures (CLS), was determined using dihydroethidium (DHE) staining. Calnexin mutants *mini_cnx1* (mini, SP18344), *lumenal_cnx1* (lum,SP18346), *lumenalTM-cnx1* (lum-tm, SP18348), *Δhcd_cnx1* (hcd, SP18350), *mini_cnx1*+*lumenalTM-cnx1* (lum-tm + mini, SP18282) were evaluated in comparison to WT calnexin (WT,SP18342), and to the vacuolar-proteases deficient strain *isp6psp3* (SP18417). **(A)** Cells were grown in minimal media (EMM) until exponential phase (0.3–0.4 OD/ml), washed twice in water, and then starved for nitrogen in EMM minus nitrogen (EMM-N). Culture samples were taken at exponential phase (EP) and days 1, 4 and 6 of nitrogen-starvation, stained with DHE (10 μM) for 30 min at 30°C. Sample of 10000 cells were analyzed by flow cytometry (BD FACSAria). **(B)** Cells were grown in CLS medium [EMM supplemented with all amino acids at 112 mg/L (EMMC)]. Culture samples were taken at EP and days 1, 4 and 7 of stationary phase and processed as above for DHE staining. For each-bar graph, data represent means ± s.e.m. of DHE-positive cells per viable cells (as determined by Phloxin B staining, see M&M). Each experiment was repeated at least three times.

Exacerbated ROS production is a common feature of apoptotic cell death [2,6,7]. We investigated whether the cell death of Cnx1p mutants involves the accumulation of ROS. Cells expressing wild-type and truncated versions of Cxn1p were submitted to nitrogen depletion and chronological aging. Samples were taken at different time points and stained with DHE or Phloxin B to assess ROS presence and cell viability, respectively. Data from flow cytometry were used to calculate the ratio of ROS-positive cells per live cells (see Materials and Methods). The number of cell accumulating ROS during exponential phase was less than 5% and not noticeably different between *cnx1* mutant cells and WT cells (Fig. 7A, B). Remarkably, during nitrogen depletion, *mini_cnx1* showed a continuous increase in ROS-positive cells, reaching 100% for the low number of remaining living cells in day 5 (Fig. 7A). Interestingly, ROS accumulation in *mini_cnx1* cells was drastically reduced by co-expressing *lumenalTM*_*cnx1* (Fig. 7A). However, the *Δhcd_cnx1* mutant also exhibited a high levels of ROS-positive cells, nevertheless viability was reduced compared to WT but not as low as *mini_cnx1*. This suggests that *Δhcd_cnx1* mutant is able to better cope with the ROS or that the ROS are not the only factor causing death.

Since *mini_cnx1* cells die rapidly in stationary phase, ROS presence was also detected in aging cultures. As shown in Fig. 7B, the level of ROS-positive cells in the *mini_cnx1* culture is about four-fold higher than in wild-type cells at day 1 of aging. The level of ROS-positive cells in *mini_cnx1*+*lumenalTM*_*cnx1* cells was lower than in *mini_cnx1* cells alone for day 1. However, at later times of the aging culture (days 4 and 7) the amount of ROS-positives cells for the *cnx1* mutants is not significantly different than wild type. The level of ROS in *mini_cnx1* cells decreases by the co-expression of lumenalTM_Cnx1p and corresponds also to an increase in viability under both, nitrogen-starvation (Fig. 5B) and aging conditions (Fig. 5D). Other apoptosis markers such as metacaspase activation, nuclear fragmentation, and Annexin V labeling were analyzed as we did previously [29,30]. The results were negative or not conclusive because of intrinsic fluorescence in these growth conditions (data not shown). Nevertheless, these observations suggest that the high presence of the apoptosis marker ROS in *mini_cnx1* cells could contribute to cell death.

### Calnexin is involved in cell wall metabolism

**Fig 8:**
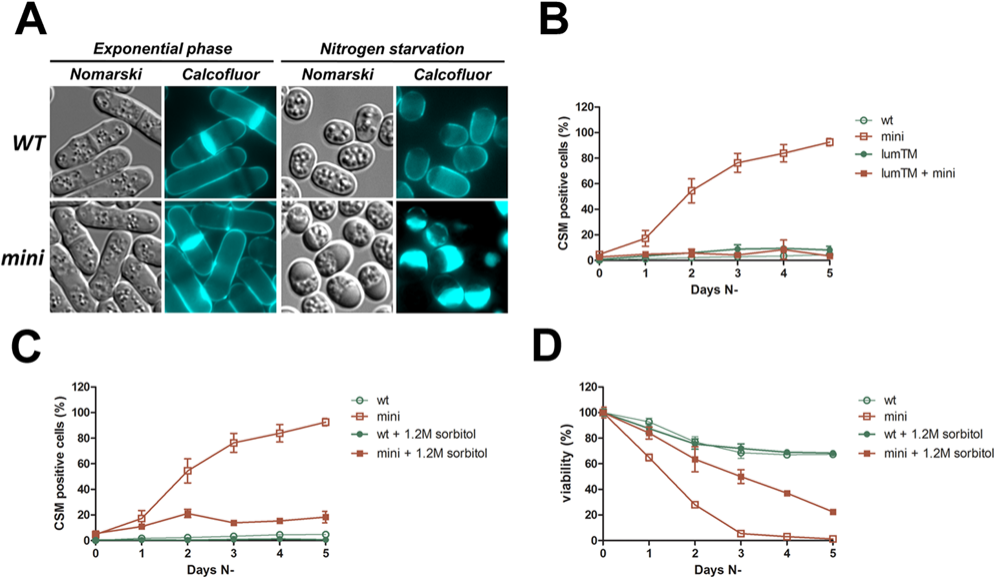
Calnexin is involved in cell wall metabolism. **(A, B)** Analysis of Calcofluor stainable material (CSM)-positive cells for the calnexin mutants *mini_cnx1* (mini, SP18344), *lumenalTM_cnx1* (lumTM, SP18348), and *mini_cnx1* + *lumenalTM_cnx1* (lumTM + mini, SP18285), under nitrogen-starvation stress in comparison to a WT strain (WT, SP18342). The presence of CSM-positive cells **(C)** and viability **(D)** of *mini_cnx1* (mini SP18344) mutant under nitrogen-starvation stress with or without 1.2 M sorbitol was analyzed in comparison to a WT strain (WT, SP18342). About 300 starved cells were observed in three independent experiments by fluorescence microscopy.

Fluorescence-microscopy observations with the fluorescent Calcofluor-white marker revealed that *mini_cnx1* cells under nitrogen starvation exhibit severe cell-wall defects. Under these conditions, *mini_cnx1* cells accumulate large aggregates of Calcofluor-stainable material, that here we called CSM. This phenotype raised the possibility that a defect in cell wall integrity during the adaptive response to nitrogen restriction could be another explanation for the rapid loss of viability of *mini_cnx1* cells. To study this particular phenotype, we analyzed the formation of CSM in different *cnx1* truncation mutants for several days following the shift to the nitrogen-free medium. As shown in Fig. 8A, CSM were absent in exponentially growing *mini_cnx1* cells cultured in minimal medium containing nitrogen. However, CSM accumulate in *mini_cnx1* cells starved for nitrogen, and are usually found in one pole of the cell. Starting with a standard morphology at exponential phase, the number of CSM-positive cells increases dramatically in the *mini_cnx1* culture during nitrogen starvation compared to WT and *lumenalTM_cnx1* (Fig. 8B). Remarkably, co-expression of *lumenalTM_cnx1* and *mini_cnx1* reverses this aberrant phenotype to WT levels (Fig. 8B). This suggests that the CSM phenotype observed with *mini_cnx1* during nitrogen starvation is recessive to the functions encoded by *lumenalTM_cnx1*.

Sorbitol is an osmotic stabilizer that reverses several cell-wall phenotypes in yeast [36]. As shown in Fig. 8C, the addition of sorbitol to the medium lacking nitrogen prevents the accumulation of CSM in *mini_cnx1* cells. This observation further supports the notion that *mini_cnx1* cells are defective in cell-wall metabolism when under the stress of nitrogen depletion. Interestingly, the decrease in CSM correlates with an increase in viability of *mini_cnx1* cells, albeit without restoring viability to WT levels (Fig. 8D). For instance, the viability of *mini_cnx1* cells in sorbitol was of 40% at 4 days of nitrogen depletion and that of WT cells was of about 70%, and after 5 days the viability *mini_cnx1* cells was of about 20% while that of WT remained at about 70%. That sorbitol did not restore viability to WT levels indicates that other cells processes are also involved in cell death of *mini_cnx* cells.

**Fig 9:**
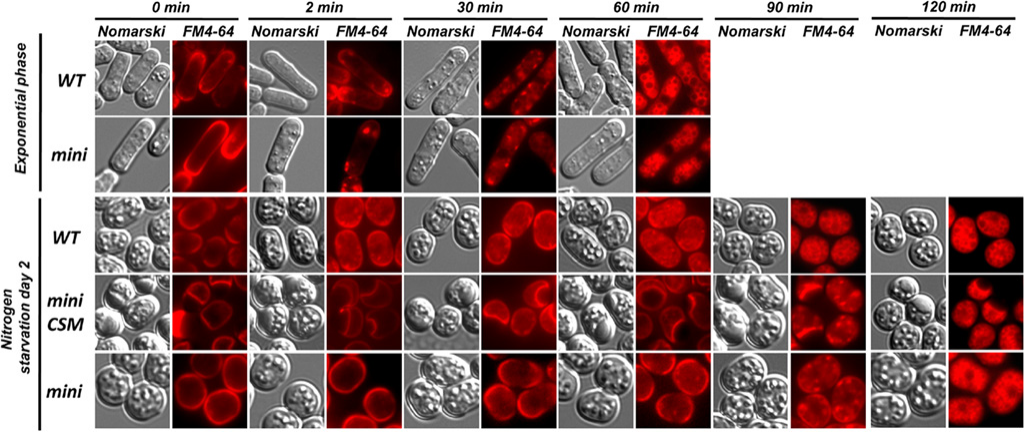
Internalization of FM4–64 is delayed in *mini_cnx1* cells under nitrogen starvation. Time course of FM4–64 uptake analyzed by fluorescence microscopy for the WT and *mini_cnx1* strains. Cells were grown in EMM to mid-logarithmic phase or in EMM-N medium to induce nitrogen starvation for 2 days and stained with FM4–64 to monitor the endocytic flux. The rate of endocytosis was followed by fluorsence microscopy with FM4–64, and cell images were recorded at the indicated times.

Endocytosis-deficient mutants exhibit cell-wall defects [55]. The rate of endocytosis can be monitored with the fluorescent, vital die FM4–64 that stains the vacuoles entering the cell via the endocytic pathway [39]. To investigate the possibility that endocytosis could be affected in *mini_cnx1* cells, we compared the kinetics of the FM4–64 uptake in *mini_cnx1* and WT cells during exponential growth and in nitrogen-depletion conditions (Fig. 9). The rate of uptake of FM4–64 was similar in exponentially growing *mini_cnx1* and WT cells. Fluorescence-microscopy showed that during the 2 first minutes the FM4–64 remains at the cell surface, then it labels small internal vesicular patches within 30 minutes, and finally reaches the vacuoles after 60 minutes. In contrast, in nitrogen-starved cells, a noticeable delay in the rate of FM4–64 uptake was observed in *mini_cnx1* cells compared to WT. While FM4–64 reaches the vacuole within 90 minutes in WT cells, in *mini_cnx1* cells the die still has not reached the vacuole. Instead, in *mini_cnx1* cells the FM4–64 reaches the vacuole by the 120 minutes time point, indicating that endocytosis is slowed down but not blocked. Remarkably, *mini_cnx1* cells exhibited the same reduced endocytosis rates independently of the CSM presence.

Taken together, these results indicate that under nitrogen-depletion *mini_cnx1* cells are affected in cell-wall metabolism and exhibit a reduction of the rate of endocytosis, being likely that these two phenotypes are linked. While under nitrogen starvation, the cell-wall phenotype correlates with the loss of viability of *mini_cnx1* cells, this defect might not be the only cause for death as sorbitol does not restore viability to WT levels.

## Discussion

The cellular role of calnexin is beyond its chaperone activity. Previous studies in mammalian cells and fission yeast have demonstrated its involvement in apoptosis induced by ER stress or caused by inositol starvation [26,28,29,30,56]. Furthermore, the cleavage of calnexin was proposed to participate in the transductional signaling of apoptosis in mammalian cells [57] as well as under inositol starvation in *S*. *pombe* [30]. Here we show that *S*. *pombe* calnexin (Cnx1p) undergoes proteolytic processing during nitrogen starvation and stationary phase. Under these conditions, Cnx1p is also required for survival and cell-wall integrity.

Our data demonstrate that the processing of Cnx1p is dependent on the activity of the vacuolar proteases Isp6 and Psp3. At this point, we cannot exclude the possibility that the effect of Isp6 and Psp3 could be indirect, and that other proteases could be more directly involved. However, no inhibition of the cleavage of Cnx1p was observed in the background of other protease knockouts tested.

The sub-cellular localization of Cnx1p changes from the ER during exponential growth to the vacuole in stationary phase or under nitrogen starvation. Thus, making it possible that the processing of Cnx1p takes place in the vacuole. Calnexin in *S*. *pombe* is cleaved in its lumenal domain, which is accessible for the active Isp6p and Psp3p or other putative proteases in the lumen of the vacuole. Remarkably, the trafficking of Cnx1p-Venus to the vacuole is blocked in the *isp6Δpsp3Δ* double mutant, thus raising the possibility that inhibition of the processing of Cnx1p in this background could be due to defective targeting to the vacuole. Mammalian calnexin, CNX1, is also proteolytically processed, however it is cleaved within its cytosolic tail by caspase 8 in human cells [58] or presumably by caspase 3 or 7 in a murine model [57].

ER proteins can be detected in the vacuole of *S*. *cerevisiae* after rapamycin treatment (a drug simulating nutrient starvation by inactivating the Tor pathway), carbon starvation, or nitrogen depletion [59]. Under these conditions, the transport of these ER proteins to the vacuole is mediated by autophagy [59,60]. These observations may explain the trafficking and processing of calnexin in *S*. *pombe* under nitrogen starvation or chronologically aging cultures, when portions of the ER containing Cnx1p are sorted directly to the vacuole, or used in phagosome formation to be finally fused with the vacuolar contents. These possibilities are supported by our observation that other ER-resident proteins (Sec61p and an ER-retained GFP) localize to the vacuole under nitrogen starvation and in stationary phase.

Here, we also show that the processing of Cnx1p is significantly reduced in autophagy-impaired strains, which correlates with the inhibition of Cnx1p transport to the vacuole. Thus, the processing of Cnx1p and its transport to the vacuole are related. However, the cleavage of Cnx1p is not entirely blocked in the *atgΔ* background suggesting that, in addition to autophagy, other pathways appear to be involved in the processing of Cnx1p. Thus, we tested the genetic inhibition of the Tor1, Sck2 and Sty1 nutrient-signaling pathways [9,51,52]. Blockage of these pathways did not show a significant defect in processing of Cnx1p (data not shown). On the other hand, preliminary experiments performed with *vps34Δ* and *sar1–1* strains (mutations blocking ER-Golgi-vacuole anterograde transport [61,62], suggest that these gene products could also be involved in the sorting of Cnx1p to the vacuole.

The relocation of calnexin has been also described in mammalian cells. Phosphorylation and palmitoylation are two mechanisms suggested to regulate the cellular distribution of mammalian calnexin [63,64,65]. Serine phosphorylation of CNX1 by CK2 reduces its interaction with PACS-2 (a sorting protein involves in ER-trafficking), consequently promoting the accumulation of CNX1 in the ER and the mitochondria-associated membrane domains (MAM) [63]. In addition, palmitoylation allows calnexin to interact with the ribosome-translocon complex in the ER, and reduced levels of palmitoylation after a tunicamycin treatment promote the retention of mammalian calnexin in the rough ER by interaction with ERp57 [64,65].

While it is clear that Cnx1p is cleaved into two moieties, the cellular role of this processing appears less obvious. To address the physiological role of its processing, we attempted to create a non-cleavable version of Cnx1p by making several point and deletion mutants overlapping the processing sites determined by N-terminal sequencing (see S4 Fig.). Intriguingly, all mutants were nevertheless processed. These observations indicate that the cleavage site of Cnx1p can be relatively non-specific as it occurs within a flexible region of the protein. Moreover, as the cleavage occurs in all Cnx1p tested mutants, these results further point to the cellular importance of the processing of calnexin in *S*. *pombe*. Proteolytic processing of proteins has been described in other adaptive stress responses. For example, in *S*. *cerevisiae*, clipping of the histone H3 N-terminus has been associated with gene activation in stationary phase, and this processing is mediated by the Prb1 vacuolar protease [66]. Also, in *S*. *pombe*, Sre1p is activated by cleavage in response to sterol depletion, and also stimulates transcription of genes required for adaptation to hypoxia [67].

Our current results demonstrate that cells under nitrogen starvation and chronological aging require both the L_Cnx1p and S_Cnx1p cleavage products for survival. Individual expression of lumenalTM_Cnx1p or mini_Cnx1p results in a remarkably loss of cell viability. Mimicking the processing of Cnx1p by co-expressing both parts of Cnx1p as lumenalTM_Cnx1p and mini_Cnx1p restores full viability. Previous studies from our laboratory have established that different domains of Cnx1p play different roles in stress-induced apoptosis. The cytosolic domain exerts an anti-apoptotic role under inositol starvation whereas the lumenal portion of Cnx1p reduces apoptotic cell death due to ER-stress induced with tunicamycin [29,30]. Thus, the production of two moieties from a single Cnx1p molecule may provide the simultaneous addition of different cellular functions, at least one for each Cnx1p fragment, under conditions of aging cultures or nitrogen starvation. Each Cnx1p moiety may act as a scaffold in the assembly of protein complexes involved in the response to the nitrogen-starvation stress and in chronological aging. While we favor the latter explanation, an alternative hypothesis could be that the cleavage of Cnx1p could lead to the loss of a toxic function under nitrogen starvation or stationary phase.

A recent report has assigned a role for one of the released fragments of CNX1 in human cells. Lakkaruju and van der Goot [58], showed that CNX1 is cleaved specifically in response to EGF and the released moiety activates STAT-3-transcription-mediated by interaction with its inhibitor PIAS3. Moreover, in mammals the cytosolic tail of calnexin operates as scaffold for the ER transmembrane protein Bap31 to be cleaved by caspase-8 after tunicamycin stress [26]. In the case of Cnx1p in *S*. *pombe*, as the L_Cnx1p moiety contains the lectin, chaperone and calcium-binding activities, this cleavage product could provide to the vacuole these functions important for survival under stress conditions. Whereas, the S_Cnx1p as it contains the TM and the cytosolic tail, this cleavage product could participate in scaffolding of complexes important for signaling and/or in inter-organellar communication (i.e. with the ER, the nucleus, and or mitochondria).

The yeast cell wall is essential both in the normal life cycle and under environmental stress conditions to maintain cell shape and cell integrity (reviewed in [68]). Here we observe severe cell-wall defects defined as Calcofluor-stainable material (CSM) in *mini_cnx1* cells cultured under nitrogen depletion. It was previously reported that endocytosis-deficient mutants exhibit cell-wall defects [55]. Interestingly, *mini_cnx1* cells exhibited a slowdown in the rate of endocytosis, suggesting for the first time the involvement of calnexin in this process. In budding yeast, cells deleted for *CNE1*, its calnexin homologue, showed an increased level of cell-wall components 1,6-*β*-glucans [69] and chitin [70], as well as an increase in cell-wall thickness [69]. One could speculate that *mini_cnx1* cells either are unable to remodel their cell wall as evidenced by the endocytosis defect we observed during nitrogen starvation, or from a general defect in the cell-wall integrity that constantly activates the synthesis of new cell-wall components. It is noteworthy that previous reports from our laboratory have shown that *mini_cnx1* cells exhibit have high levels of secretion of certain proteins [71,72]. Thus, an imbalance between endocytosis and secretion could explain the cell-wall defects in *mini_cnx1* cells.

From yeast to mammals, the production of ROS is found in numerous situations affecting cell viability, leading to death by apoptosis and/or necrosis [6,7,8,73]. In yeast, overproduction of ROS has been reported under starvation of specific amino acids and in aging cells [74,75,76]. Here we show that the *cnx1* mutants tested, in particular *mini_cnx1* cells produce high levels of ROS under nitrogen starvation and in aging cultures, suggesting that ROS may be involved in the death of *mini_cnx1* mutant. Interestingly however, a large proportion of *Δhcd_cnx1* cells were ROS-positive, nonetheless they exhibit better viability than *mini_cnx1* cells. This suggests that on one hand, *Δhcd_cnx1* cells can better cope with ROS or, other causes could lead to the death of *mini_cnx1* cells, which may include cell-wall defects. Recent reports have shown that autophagy and apoptosis can be activated simultaneously by the same stimuli [77,78]. It is proposed that autophagy can protect the cell from some effects triggered by apoptosis pathways. In this context, as it is involved in both processes, Cnx1p could be a link for the intersection of these pro-death and pro-survival pathways.

The results in mammalian systems together with ours in *S*. *pombe* indicate that the sub-cellular localization of calnexin responds to diverse stimuli. Both in mammals and in fission yeast, calnexin is processed in life-death determining situations. These observations suggest that the multiple roles of calnexin depend on its sub-cellular localization, on its proteolytic cleavage, and these processes are coordinated with diverse responses determining cell fate. The use of a simple model such as *S*. *pombe* should assist in further elucidating the manifold cellular roles of calnexin.

## Supporting Information

S1 Fig ER-residing proteins are targeted to vacuole during nitrogen starvation and stationary phase. The localization of the artificial ER lumenal marker Venus-ADEL and the translocon α (alpha) Sec61 subunit fused to GFP (Sec61-GFP) were used as a control to assess the level of ER trafficking to the vacuole. The strains *Δcnx1*::*his3* ^*+*^ *+* pREP41*cnx1-Venus* (SP19201), SP248+pREP42-Venus-ADEL (SP19242) and SP248+pEG3Sec61-GFP (SP19245) were grown in EMM to exponential phase, then maintained until stationary phase for 3 days, or shifted to EMM-N medium to induce nitrogen starvation (24h). Cells were analyzed by fluorescence microscopy for the ER localization, FM4–64 was used as a marker for the vacuole, and Hoechst33342 was used as a marker for the nucleus. Nomarski bright-field microscopy was used to monitor cell morphology. Merged images were used to determine colocalization.
(TIF)

S2 Fig Growth rate and survival rate of Cnx1p mutants. Growth rate **(A, B)** and survival rate **(C, D)** of strains *mini_cnx1* (mini, SP18344), *lumenal_cnx1* (lum, SP18346), *lumenalTM_cnx1* (lumTM, SP18348), *Δhcd_cnx1* (hcd, SP18350), *lumenalTM_cnx1* + *mini_cnx1* (lumTM + mini, SP18285), *isp6Δpsp3Δ* (SP18340) and WT control cells (wt, SP18342) cultured in EMM **(A, C)** or EMM supplemented with all the amino acids, except those required for selection (EMMC) **(B, D)**. Cells from freshly streaked plates were grown o/n in EMM or EMM supplemented with all the amino acids, except those required for selection (EMMC) to OD_600_ 0.5–1, diluted at 0.1 OD_600_ (time 0h) in fresh medium and grown for 48 hours. Every 6 hours during this time, the OD_600_ of each culture was taken to monitor the growth rate of each strain in EMM **(A)** or EMMC **(B)**. At each time point, an aliquot of cells from each culture were also serially-diluted and plated on the respective EMM or EMMC plates to assess the survival rate. CFU were counted after incubating the plate at 30°C for 5 days. The survival rate in EMM **(C)** and EMMC **(D)** was determined by dividing the CFU obtained at each time point to the number of CFU at time 0 h. Each experiment was repeated at least twice.
(TIF)

S3 Fig Analysis of the cleavage of Cnx1-Venus fusions. **(A)** Strains *cnx1*::*his* ^*+*^ *+* pREP41*lumenalTM_cnx1-Venus* (SP19207), pREP41*lumenal_cnx1-Venus* (SP19174), pREP41*Δhcd_cnx1-Venus* (SP19211), pREP41*mini_cnx1-Venus* (SP19212) and pREP41*cnx1p-Venus* (control, SP19201) were grown in EMM to mid-logarithmic phase. The culture was split into two, half was maintained until stationary phase for 3 days, and the other half was shifted to EMM-N medium to induce nitrogen starvation (24h). Cell samples were taken and analyzed by immunoblotting. Cnx1-Venus and tubulin (loading control) were detected with anti-Cnx1p (α-Cnx1p) or anti-tubulin (α-Tubulin), respectively. NS, non-specific band. **(B)** Strains pREP41*mini_cnx1-Venus* (SP19212) and pREP41*cnx1p-Venus* (control, SP19201) were processed as above, and analyzed by immunoblotting using anti-GFP. The presence or absence of Isp6p and Psp3p is indicated by + (plus) or—(minus) signs, respectively.
(TIF)

S4 Fig Location of cleavages site of calnexin. To determine where the cleavage occurs in Cnx1p, cells expressing a Cnx1-Venus fusion were grown to stationary phase, harvested and lysed. The Cnx1p C-terminal cleavage product (fused to Venus) was immunoprecipitated with GFP-TRAP (Chromotek, Germany) and subjected to N-terminal Edman sequencing. Following the sequencing results, we created an Ala-substitution mutant of the cleavage site and assessed the Cnx1p processing. Since Cnx1p was nevertheless processed, we thus determined the N-terminal sequence of the Ala-substitution Cnx1-Venus mutant. Yet again, the double-Cnx1p mutant was processed. The same approach was repeated twice more, and the quadruple-Cnx1p mutant was nevertheless processed. The four cleavage sites identified (Lys432/Ser433, Asp446/Glu447, Lys453/Glu454 and Glu480/Thr481) in multiple experiments are represented by arrows in the sequence, with the first sequenced residue is shown in bold. Ala-substitution mutant are indicated with * above the sequence. Underline residues represent the transmembrane domain. Explain more of this experiment and also talk about deletion mutants.
(TIF)

S1 Table Strains used in this study. (DOCX)

S2 Table Doubling time of calnexin mutant strains. (XLSX)

S1 File Supplementary Materials and Methods. (DOCX)
