# Supplementary figures and images for: Calnexin Is Essential for Survival under Nitrogen Starvation and Stationary Phase in Schizosaccharomyces pombe
Source: PLoS One. 2015 Mar 24;10(3):e0121059. doi: 10.1371/journal.pone.0121059 (PMC4372366; doi:10.1371/journal.pone.0121059)

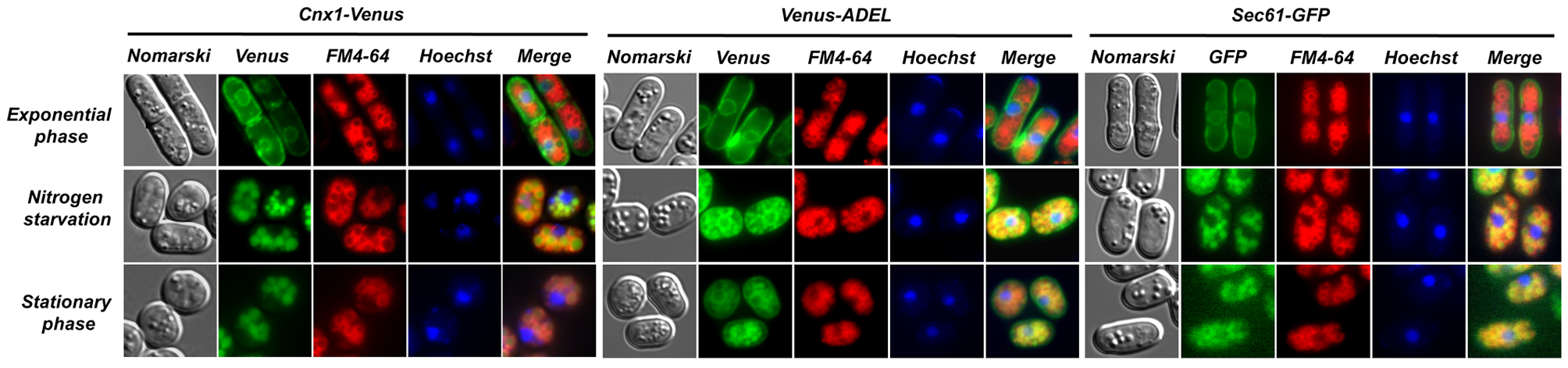

Supplement: S1 Fig — The localization of the artificial ER lumenal marker Venus-ADEL and the translocon α (alpha) Sec61 subunit fused to GFP (Sec61-GFP) were used as a control to assess the level of ER trafficking to the vacuole. The strains Δcnx1::his3 + + pREP41cnx1-Venus (SP19201), SP248+pREP42-Venus-ADEL (SP19242) and SP248+pEG3Sec61-GFP (SP19245) were grown in EMM to exponential phase, then maintained until stationary phase for 3 days, or shifted to EMM-N medium to induce nitrogen starvation (24h). Cells were analyzed by fluorescence microscopy for the ER localization, FM4–64 was used as a marker for the vacuole, and Hoechst33342 was used as a marker for the nucleus. Nomarski bright-field microscopy was used to monitor cell morphology. Merged images were used to determine colocalization. (TIF) [file pone.0121059.s001.tif]

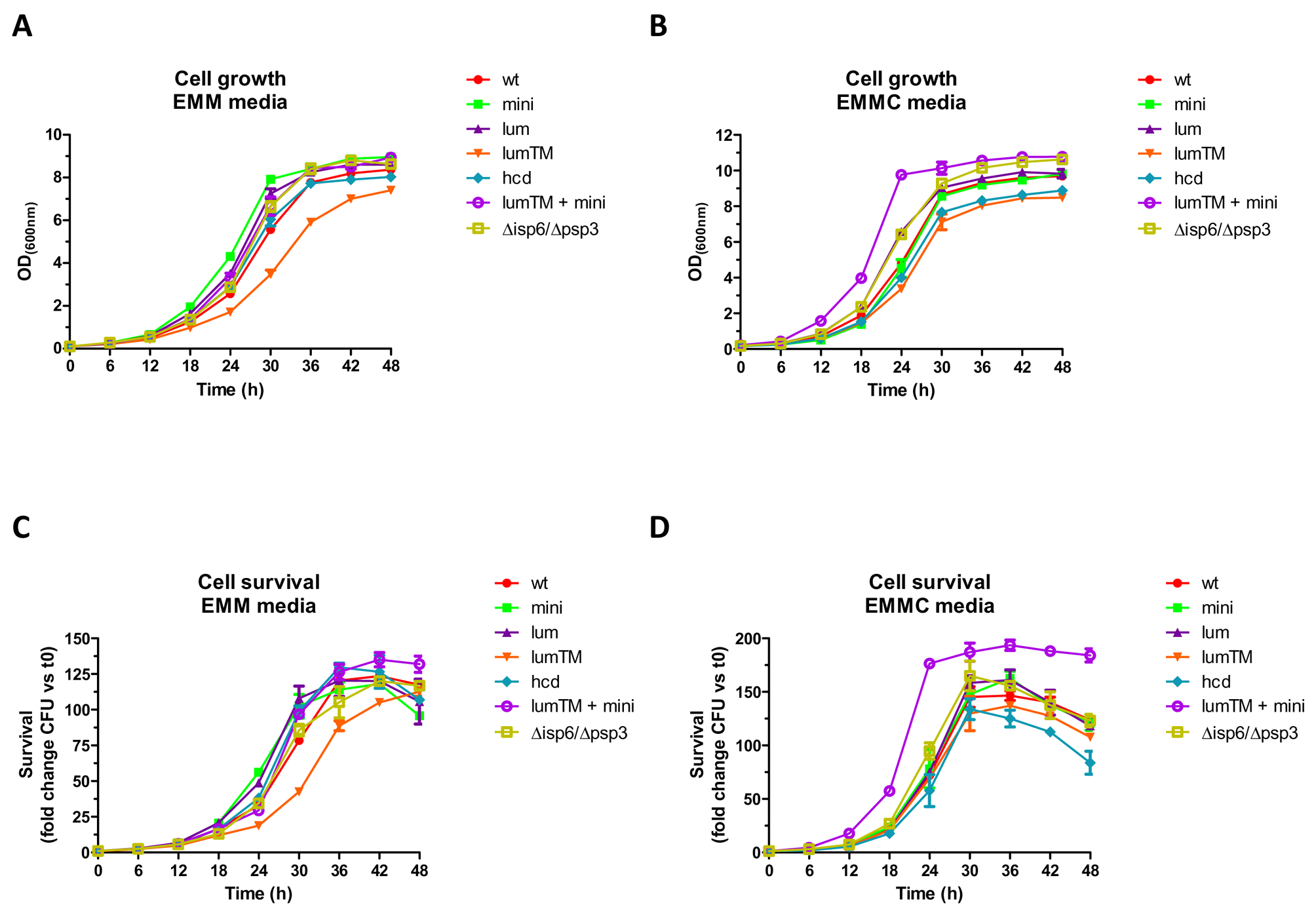

Supplement: S2 Fig — Growth rate (A, B) and survival rate (C, D) of strains mini_cnx1 (mini, SP18344), lumenal_cnx1 (lum, SP18346), lumenalTM_cnx1 (lumTM, SP18348), Δhcd_cnx1 (hcd, SP18350), lumenalTM_cnx1 + mini_cnx1 (lumTM + mini, SP18285), isp6Δpsp3Δ (SP18340) and WT control cells (wt, SP18342) cultured in EMM (A, C) or EMM supplemented with all the amino acids, except those required for selection (EMMC) (B, D). Cells from freshly streaked plates were grown o/n in EMM or EMM supplemented with all the amino acids, except those required for selection (EMMC) to OD600 0.5–1, diluted at 0.1 OD600 (time 0h) in fresh medium and grown for 48 hours. Every 6 hours during this time, the OD600 of each culture was taken to monitor the growth rate of each strain in EMM (A) or EMMC (B). At each time point, an aliquot of cells from each culture were also serially-diluted and plated on the respective EMM or EMMC plates to assess the survival rate. CFU were counted after incubating the plate at 30°C for 5 days. The survival rate in EMM (C) and EMMC (D) was determined by dividing the CFU obtained at each time point to the number of CFU at time 0 h. Each experiment was repeated at least twice. (TIF) [file pone.0121059.s002.tif]

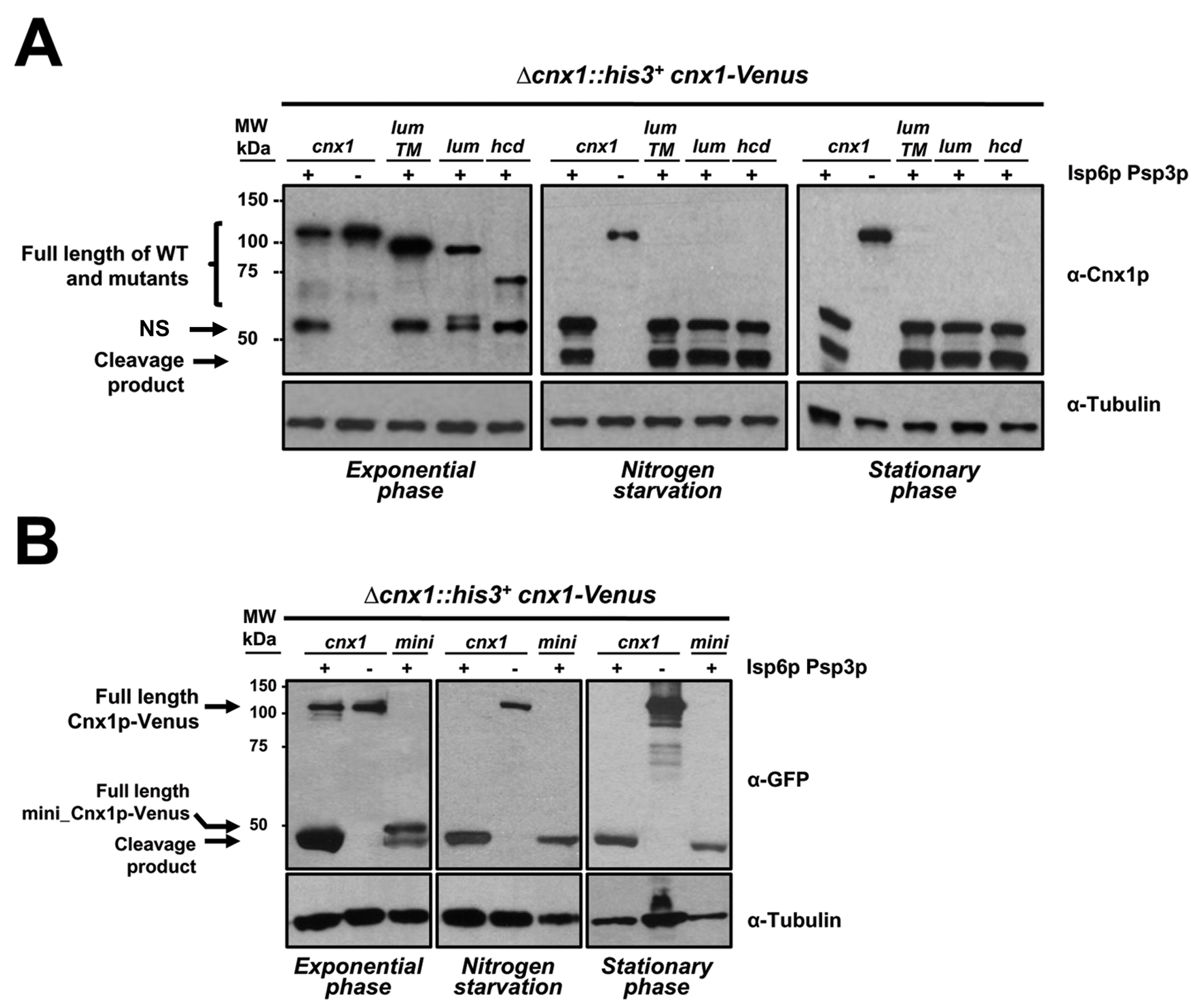

Supplement: S3 Fig — (A) Strains cnx1::his + + pREP41lumenalTM_cnx1-Venus (SP19207), pREP41lumenal_cnx1-Venus (SP19174), pREP41Δhcd_cnx1-Venus (SP19211), pREP41mini_cnx1-Venus (SP19212) and pREP41cnx1p-Venus (control, SP19201) were grown in EMM to mid-logarithmic phase. The culture was split into two, half was maintained until stationary phase for 3 days, and the other half was shifted to EMM-N medium to induce nitrogen starvation (24h). Cell samples were taken and analyzed by immunoblotting. Cnx1-Venus and tubulin (loading control) were detected with anti-Cnx1p (α-Cnx1p) or anti-tubulin (α-Tubulin), respectively. NS, non-specific band. (B) Strains pREP41mini_cnx1-Venus (SP19212) and pREP41cnx1p-Venus (control, SP19201) were processed as above, and analyzed by immunoblotting using anti-GFP. The presence or absence of Isp6p and Psp3p is indicated by + (plus) or—(minus) signs, respectively. (TIF) [file pone.0121059.s003.tif]

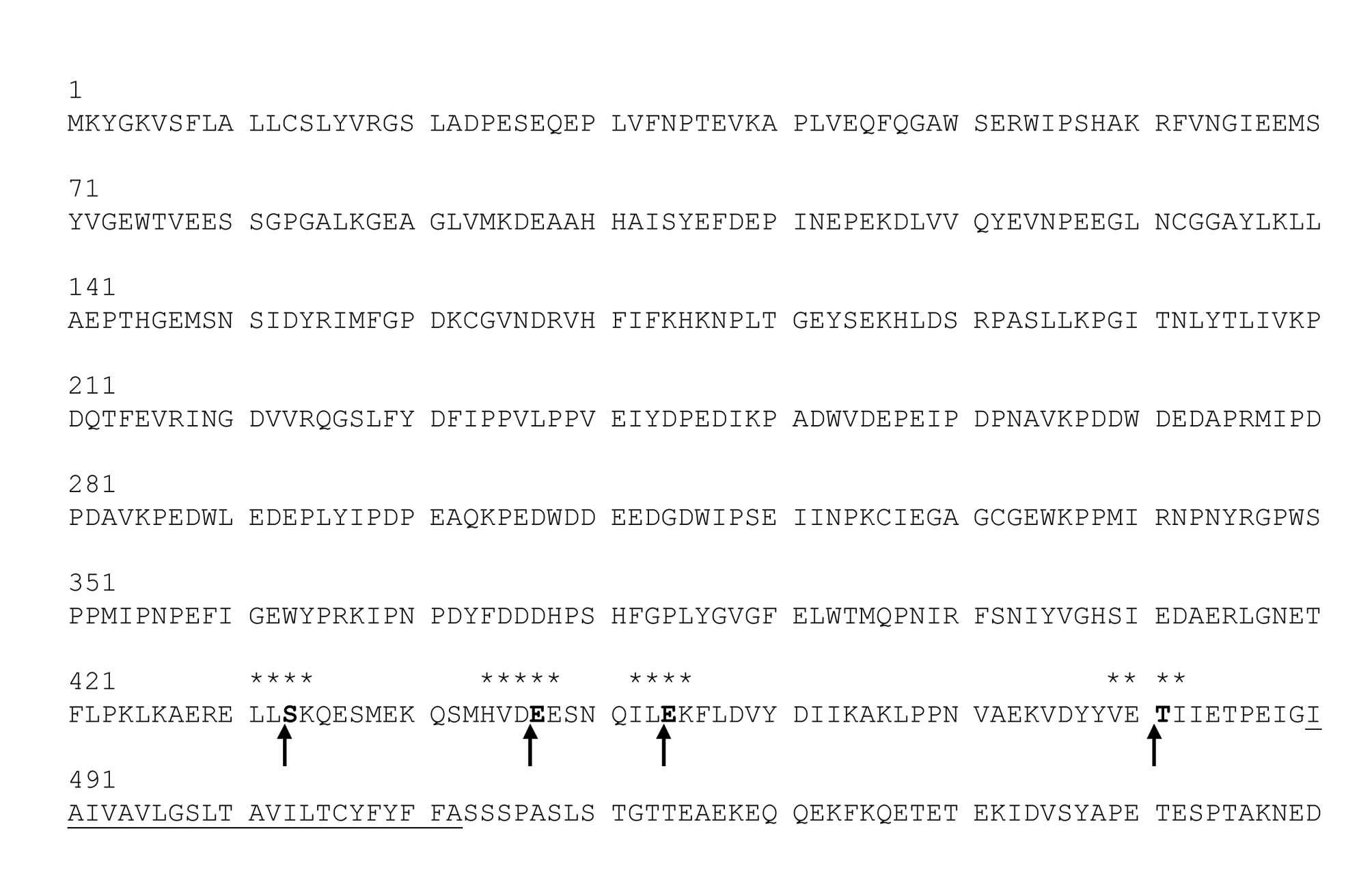

Supplement: S4 Fig — To determine where the cleavage occurs in Cnx1p, cells expressing a Cnx1-Venus fusion were grown to stationary phase, harvested and lysed. The Cnx1p C-terminal cleavage product (fused to Venus) was immunoprecipitated with GFP-TRAP (Chromotek, Germany) and subjected to N-terminal Edman sequencing. Following the sequencing results, we created an Ala-substitution mutant of the cleavage site and assessed the Cnx1p processing. Since Cnx1p was nevertheless processed, we thus determined the N-terminal sequence of the Ala-substitution Cnx1-Venus mutant. Yet again, the double-Cnx1p mutant was processed. The same approach was repeated twice more, and the quadruple-Cnx1p mutant was nevertheless processed. The four cleavage sites identified (Lys432/Ser433, Asp446/Glu447, Lys453/Glu454 and Glu480/Thr481) in multiple experiments are represented by arrows in the sequence, with the first sequenced residue is shown in bold. Ala-substitution mutant are indicated with * above the sequence. Underline residues represent the transmembrane domain. Explain more of this experiment and also talk about deletion mutants. (TIF) [file pone.0121059.s004.tif]
