# Supplementary material for: Calnexin Is Essential for Survival under Nitrogen Starvation and Stationary Phase in Schizosaccharomyces pombe
Source: PLoS One. 2015 Mar 24;10(3):e0121059. doi: 10.1371/journal.pone.0121059 (PMC4372366; doi:10.1371/journal.pone.0121059)
Supplement: S1 Table — (DOCX) [file pone.0121059.s005.docx]

| **S1 Table. Strains used in this study** | | | |
| --- | --- | --- | --- |
| **Strain** | **Genotype** | | **Source/Reference** |
| SP247 | *h^-^* | *ade6-M210 ura4-D18 leu1-32 his3-D1* | Burke & Gould [[76](#_ENREF_76)] |
| SP248 | *h^+^* | *ade6-M210 ura4-D18 leu1-32 his3-D1* | Burke & Gould [[76](#_ENREF_76)] |
| SP18041 | *h^+^* | *ade6-M210 ura4-D18 leu1-32 his3-D1 Δisp6::ura4^+^* | This work |
| SP17174 | *h^-^* | *ade6-M210 ura4-D18 leu1-32 Δpsp3::KanMX4* | Bioneer |
| SP17319 | *h^-^* | *ade6-M210 ura4-D18 leu1-32 his3-D1 Δpsp3::KanMX4* | This work |
| SP17198 | *h^-^* | *ade6-M216 ura4-D18 leu1-32 Δsxa1::ura4^+^* | YGRC (FY13331) |
| SP17199 | *h^-^* | *ade6-M216 ura4-D18 leu1-32 Δsxa2::ura4^+^* | YGRC (FY7119) |
| SP17260 | *h^+^* | *ade6-M216 ura4-D18 leu1-32 Δcpy1::KanMX4* | Bioneer |
| SP17175 | *h^+^* | *ade6-M216 ura4-D18 leu1-32 Δpgp1::KanMX4* | Bioneer |
| SP17176 | *h^+^* | *ade6-M216 ura4-D18 leu1-32 Δyps1::KanMX4* | Bioneer |
| SP17220 | *h^+^* | *ade6-M216 ura4-D18 leu1-32 ΔSPAC3H1.05::KanMX4 (caax prenyl protease)* | Bioneer |
| SP17221 | *h^+^* | *ade6-M216 ura4-D18 leu1-32 ΔSPBC18A7.01::KanMX4 (X-pro dipeptidase)* | Bioneer |
| SP17222 | *h^+^* | *ade6-M216 ura4-D18 leu1-32 ΔSPBC16G5.09::KanMX4 (serine carboxipeptidase)* | Bioneer |
| SP17223 | *h^+^* | *ade6-M216 ura4-D18 leu1-32 ΔSPACUNK4.08::KanMX4 (dipeptidyl peptidase)* | Bioneer |
| SP18417 | *h^-^* | *ade6-M210 ura4-D18 leu1-32 his3-D1 Δpsp3::KanMX4 Δisp6::ura4^+^(FOA) + pREP41 + pSLF272* | This work |
| SP18419 | *h^-^* | *ade6-M210 ura4-D18 leu1-32 his3-D1 Δpsp3::KanMX4 Δisp6::ura4^+^(FOA) + pREP41isp6-HA + pSLF272* | This work |
| SP18421 | *h^-^* | *ade6-M210 ura4-D18 leu1-32 his3-D1 Δpsp3::KanMX4 Δisp6::ura4^+^(FOA) + pREP41 + pSLF272psp3-HA* | This work |
| SP18423 | *h^-^* | *ade6-M210 ura4-D18 leu1-32 his3-D1 Δpsp3::KanMX4 Δisp6::ura4^+^(FOA) + pREP41isp6-HA + pSLF272psp3-HA* | This work |
| SP18147 | *h^+^* | *ade6-M21? ura4-D18 leu1-32 his3-D1 Δcnx1::his3^+^ + pREP42cnx1-myc + pTN54GFPatg8* | This work |
| SP18084 | *h^+^* | *ade6-M21? ura4-D18 leu1-32 his3-D1 Δatg1::KanMX4 Δcnx1::his3^+^ + pREP41cnx1-myc* | This work |
| SP18087 | *h^+^* | *ade6-M21? ura4-D18 leu1-32 his3-D1 Δatg6::KanMX4 Δcnx1::his3^+^ + pREP41cnx1-myc* | This work |
| SP18086 | *h^-^* | *ade6-M21? ura4-D18 leu1-32 his3-D1 Δatg8::KanMX4 Δcnx1::his3^+^ + pREP41cnx1-myc* | This work |
| SP18414 | *h^+^* | *ade6-M21? ura4-D18 leu1-32 his3-D1 Δatg9::KanMX4 Δcnx1::his3^+^ + pREP41cnx1-myc* | This work |
| SP18340 | *h?* | *ade6-M21? ura4-D18 leu1-32 his3-D1 Δpsp3::KanMX4 Δisp6::ura4^+^ Δcnx1::his3^+^ + pREP41cnx1-myc* | This work |
| SP18342 | *h^+^* | *ade6-M216 ura4-D18 leu1-32 his3-D1 Δcnx1::his3^+^ + pREP41cnx1^+^ + pREP42* | This work |
| SP18344 | *h^-^* | *ade6-M216 ura4-D18 leu1-32 his3-D1 Δcnx1::his3^+^ + pREP41mini_cnx1 + pREP42* | This work |
| SP18346 | *h^-^* | *ade6-M216 ura4-D18 leu1-32 his3-D1 Δcnx1::his3^+^ + pREP41lumenal_cnx1 + pREP42* | This work |
| SP18348 | *h^-^* | *ade6-M216 ura4-D18 leu1-32 his3-D1 Δcnx1::his3^+^ + pREP41lumenalTM_cnx1 + pREP42* | This work |
| SP18350 | *h^-^* | *ade6-M216 ura4-D18 leu1-32 his3-D1 Δcnx1::his3^+^ + pREP41Δhcd_cnx1 + pREP42* | This work |
| SP18282 | *h?* | *ade6-M216 ura4-D18 leu1-32 his3-D1 Δcnx1::his3^+^ + pREP41lumenal_cnx1 + pREP42mini_cnx1* | This work |
| SP18300 | *h^-^* | *ade6-M216 ura4-D18 leu1-32 his3-D1 Δcnx1::his3^+^ + pREP41lumenal_cnx1 + pREP42C-term-Venus* | This work |
| SP18285 | *h?* | *ade6-M216 ura4-D18 leu1-32 his3-D1 Δcnx1::his3^+^ + pREP41lumenalTM_cnx1 + pREP42mini_cnx1* | This work |
| SP18303 | *h^-^* | *ade6-M216 ura4-D18 leu1-32 his3-D1 Δcnx1::his3^+^ + pREP41lumenalTM_cnx1 + pREP42C-term-Venus* | This work |
| SP19201 | *h^-^* | *ade6-M216 ura4-D18 leu1-32 his3-D1 Δcnx1::his3^+^ + pREP41cnx1-Venus* | This work |
| SP19212 | *h^-^* | *ade6-M216 ura4-D18 leu1-32 his3-D1 Δcnx1::his3^+^ + pREP41mini_cnx1-Venus* | This work |
| SP19174 | *h^-^* | *ade6-M216 ura4-D18 leu1-32 his3-D1 Δcnx1::his3^+^ + pREP41lumenal_cnx1-Venus* | This work |
| SP19207 | *h^-^* | *ade6-M216 ura4-D18 leu1-32 his3-D1 Δcnx1::his3^+^ + pREP41lumenalTM_cnx1-Venus* | This work |
| SP19211 | *h^-^* | *ade6-M216 ura4-D18 leu1-32 his3-D1 Δcnx1::his3^+^ + pREP41Δhcd_cnx1-Venus* | This work |
| SP19209 | *h^-^* | *ade6-M216 ura4-D18 leu1-32 his3-D1 Δcnx1::his3^+^ Δpsp3::KanMX4 Δisp6::ura4^+^ + pREP41 cnx1-Venus* | This work |
| SP19197 | *h^-^* | *ade6-M216 ura4-D18 leu1-32 his3-D1 Δcnx1::his3^+^ Δatg1::KanMX4 + pREP41cnx1-Venus* | This work |
| SP19242 | *h^+^* | *ade6-M210 ura4-D18 leu1-32 his3-D1 + pREP42Venus-ADEL* | This work |
| SP19245 | *h^+^* | *ade6-M210 ura4-D18 leu1-32 his3-D1+ pEG3-Sec61-GFP* | This work |
