# Supplementary material for: Calnexin Is Essential for Survival under Nitrogen Starvation and Stationary Phase in Schizosaccharomyces pombe
Source: PLoS One. 2015 Mar 24;10(3):e0121059. doi: 10.1371/journal.pone.0121059 (PMC4372366; doi:10.1371/journal.pone.0121059)
Supplement: S1 File — (DOCX) [file pone.0121059.s007.docx]

**Supplementary Materials and Methods**

**Expression vectors**

The pREP42*-VenusADEL* plasmid was constructed by adding in frame the signal peptide of Bip1 (in bold in primers) protein in N-termini of the Venus to which the ADEL ER retention signal was added in C-termini. The coding sequence was amplified using the following primers. Venus-SP-Bip (5’-gccgtcgac**atgaagaagttccagctatt tagcattttaagctactttgtagctttattcctcctacctatggcttttgctagtggt**gtgagcaAgggcgaggagc-3’) containing a Sal1 (underlined) restiction site and (5’-gccggatccttaaagttcatcggcctccttgtac agctcg tccatgc-3’) containing a BamH1 (underlined) restiction site. The obtained artificial VenusADEL protein was digested with *Sal*I-*Bam*HI and cloned into the pREP42 plasmid.

The plasmid expressing the pEG3-Sec61-GFP was provided by the NBRP of the MEXT, Japan.
